# Exploring Dopamine Neurotransmitter and Silver Nanocluster (Ag_n_; *n* = 4–20) Interactions: DFT Insights for Biomedical Applications

**DOI:** 10.1021/acsomega.5c04404

**Published:** 2025-11-25

**Authors:** Ehsan Shakerzadeh, Elham Tahmasebi, Tarun Yadav, Noe Brigido Salvador, Ernesto Chigo Anota

**Affiliations:** † Chemistry Department, Faculty of Science, 48513 Shahid Chamran University of Ahvaz, Ahvaz 61357-83151, Iran; ‡ School of Advanced Engineering, 199257 University of Petroleum and Energy Studies, Dehradun 248007, India; § 3972 Benemerita Universidad Autonoma de Puebla, Facultad de Ingenieria Quimica, Ciudad Universitaria, San Manuel, Puebla, Codigo Postal 72570, Mexico

## Abstract

This study investigates the interactions between silver nanoclusters (Ag_n_; *n* = 4–20) and the dopamine neurotransmitter using density functional theory calculations. Our results demonstrate significant binding of silver nanoclusters to the amine group of the ethylamine side chain of dopamine. The adsorption energies range from −20 to −30 kcal/mol, suggesting favorable interactions. The results reveal strong binding specifically, supported by molecular electrostatic potential, quantum theory of atoms in molecules (QTAIM), and reduced density gradient analyses. The influence of water as a solvent on the interactions was also evaluated through the integral equation formalism-polarizable continuum model. The presence of water as a solvent slightly affects the stability and electronic features, notably increasing the dipole moments and solubility in polar environments. QTAIM analysis confirms the existence of dative Ag–N bonds with ionic character, characterized by low electron densities and positive Laplacians. Ab initio molecular dynamics simulations of select systems (DOP@Ag_5_ and DOP@Ag_10_) demonstrate their thermal and structural stability at 320 K, indicating their robustness under physiological conditions. Overall, this work offers insights into the adsorption mechanisms of dopamine on silver nanoclusters with potential implications for advancements in biosensing applications.

## Graphical abstract

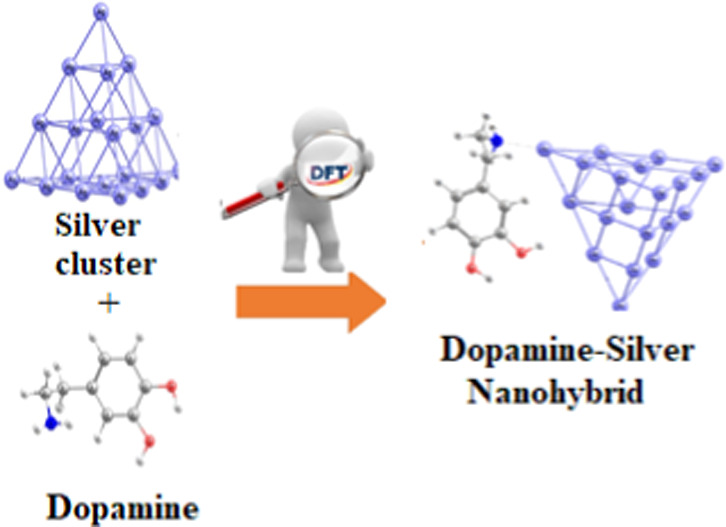

## Introduction

Medical innovations always aim toward maximizing therapeutic benefits and reducing side effects. Of the key scientific developments in the last decades, nanotechnology has become a transformative force, presenting hitherto unimaginable opportunities for medication delivery.^1,2^ Nanomaterial-engineered structures with nanoscale dimensions are the core of this revolution. The diverse chemical, biological, optical, and physical properties of these materials allow for precise modification and control over drug delivery systems.^3–8^ The role of nanomaterials in medication administration cannot be underscored as they provide us with so many benefits over conventional delivery techniques.^9^ Their tiny size offers precise delivery to particular tissues or cells, enhancing therapeutic effects while reducing systemic toxicity. Furthermore, the high surface-area-to-volume ratios of nanoparticles enable effective drug loading and release kinetics. This capability, along with tunable surface characteristics, allows medication formulations to be tailored to various therapeutic applications. Additionally, nanomaterials can overcome biological barriers such as the blood–brain barrier, enhancing drug penetration into inaccessible regions of the body.^10–13^ This ability has great potential for treatment of neurological diseases and other similar conditions for which conventional treatments remain severely limited.

There are several investigations, in which organic or inorganic nanomaterials such as carbon nanostructures and silver or gold nanoclusters interact with biomolecules, neurotransmitters, peptides, and nucleic acids. These studies explored the potential of these nanomaterials for drug delivery systems, sensing, and other applications.^14–22^ The silver nanomaterials have procured a greater extent from the research community as appropriate candidates in the biological applications because of their remarkable sets of properties that include better biocompatibility, nontoxicity, size, and shape-dependent optical and electronic properties.^23^ These substantial advantages of Ag nanoparticles (AgNPs) have led to a global interest from scientific researchers, medical professionals, and the pharmaceutical industry in the investigation and application of nanomaterials in drug delivery, biosensing, and bioimaging.^24^ Comprehending the role of AgNPs in drug delivery, biosensing, and bioimaging is not merely an issue of scientific investigation but also an essential prerequisite for improving therapeutic efficacy. Thus, the interaction of various nanoparticles with biomolecules and neurotransmitters is a burgeoning area of research, which holds significant impact for pharmaceutics, neuroscience, and nanotechnology.

A literature survey reveals several investigations including theoretical and experimental on the interaction of silver nanoparticles and biomolecules in many distinct aspects.^25–27,32^ Ando et al.^28^ developed a high-speed multicolor particle motion tracking system by using Ag, Au, and Ag–Au alloys. The silver nanoparticles were also used to explore the role of surface charge in protein interaction along with cellular cytotoxicity.^29^ Tai et al.^30^ reported on the kinetic analysis of silver nanoparticles in acidic environments and the influence of protein interactions, such as those involving bovine serum albumin, on the colloidal stability of silver nanoparticles. In another experimental investigation, the interaction of silver nanoparticles with an ionic liquid was performed through comprehensible and responsive UV–visible spectroscopic method.^31^ Silver nanoparticles were also reported as potential drug delivery carriers of antimalarial drugs to treat malaria and Covid-19 based on the computed results of the density functional theory (DFT) study.^32^ Moreover, the application of silver nanoparticles and their composite materials as drug carriers and sensors were also predicted^33–36^ in their theoretical studies. Anuar developed an electrochemical sensor based on the Pt–Ag/Gr nanocomposite to detect dopamine neurotransmitter.^37^

Dopamine neurotransmitter, which also belongs to the monoamine class of compounds like tyramine, and substantially a subclass known as catecholamines, contributes to several neural actions integrating movement, locomotion, reward, endocrine regulations, and cognition.^38^ A number of significant nervous system disorders are linked to dopamine system dysfunctions, and several of the primary medicines used to treat these disorders modify the effects of dopamine.^39^ Moreover, dopamine influences the brain systems that regulate movement, mood, and the capacity to feel both pain and pleasure. Our mental and physical health are significantly impacted by dopamine regulation.^40^ Despite many investigations on different aspects of silver nanoparticles, theoretical studies on the interaction of dopamine neurotransmitter with silver nanoparticles have not been explored in detail so far.

Among the theoretical approaches used to study adsorption on nanostructured surfaces, the atomic nanocluster model is particularly valuable because it enables direct simulation of interactions and related properties via reliable quantum-chemical calculations.^41,42^ The cluster model can be constructed by extracting a cluster from the bulk or surface, thereby reducing the problem of an infinite solid to routine calculations on molecule-like fragments.^43^ The cluster model yields rich information, since it aligns with chemical intuition. In this context, we set out to explore the adsorption properties of dopamine on the surface of silver nanoclusters. As for a simpler model, we use the silver clusters Ag_n_ (*n* = 4–20) to understand the potential use of silver nanomaterials for detecting dopamine. Understanding the adsorption mechanism of dopamine over silver nanoclusters may open up novel possibilities for advancement in biosensing applications.

## Computational Details

All calculations for are done by employing DFT available in Gaussian 09 software.^44^ Local energy minima are fully optimized without imposing any symmetry or geometrical constraints, utilizing a density functional that incorporates long-range exchange effects, specifically, the LC-BLYP functional. This functional is highly recommended for the quantitative analysis of systems involving noble metals.^45–47^ For silver atoms, the LANL2DZ basis set with an effective core potential (ECP) is employed, while the all-electron 6–311++G­(d,p) basis set is used for nonmetal atoms. The validity of the level of theory is also considered and investigated. Frequency calculations on all optimized geometries were also performed to confirm their nature as true minima. In all cases, the calculated vibrational frequencies showed no imaginary frequencies, indicating that the structures correspond to true minima on the potential energy surface. The corresponding vibrational spectra are provided in the Electronic Supporting Information (ESI) for transparency and to support the validity of our optimized geometries. The complexes DOP@Ag_n_, with even values of n (4, 6, 8, 10, 12, 14, 16, 18, and 20), contain paired electrons and thus exhibit a singlet multiplicity, which were treated by using a restricted approach. Conversely, the complexes with odd values of n (5, 7, 9, 11, 13, 15, 17, and 19) have unpaired electrons, resulting in a doublet state, and were modeled using an unrestricted approach. All the considered complexes are neutral in charge.

The influence of the solvent, specifically an aqueous solution in this study, is modeled using the integral equation formalism-polarizable continuum model (IEF-PCM).^48^ The IEF-PCM model is a quantum chemistry method that simulates solvent effects by treating the solvent as a continuous medium. By directly solving boundary integral equations, IEF-PCM provides faster, more accurate, and more stable results—especially for large systems—while avoiding some convergence issues seen in models like I-PCM. It also delivers a physically realistic portrayal of solvent polarization, improving the modeling of solvation energies.

In our study, we employed a comprehensive approach to explore the potential interaction sites. This included systematically varying the positions and orientations of DOP relative to those of the silver nanoclusters to ensure broad coverage of the surrounding spatial region for each nanocluster. Additionally, we conducted multiple independent simulations with different initial configurations. This strategy improved our sampling of the conformational space, allowing us to capture a diverse array of local minima and thereby increase the likelihood of identifying the most stable DOP@Ag_n_ complex. The adsorption energy of this most stable complex was calculated using the following equation

1 $$E_{ads}=E_{DOP@{Ag}_{n}}-\left(E_{DOP}+E_{{Ag}_{n}}\right)$$

where the first term $E_{DOP@{Ag}_{n}}$ stands for the total energy of DOP@Ag_n_ nanohybrids; the second and third terms stand for the total energy of isolated dopamine and silver nanocluster, respectively.

## Results and Discussion

The conformational and vibrational spectroscopic investigations of dopamine neurotransmitter were reported in our earlier investigation, where 15 low-lying energy conformers of dopamine neurotransmitter are found.^33^ Herein, the interaction of the most stable conformers of dopamine neurotransmitter with the silver clusters Ag_n_ (*n* = 4–20) is studied to unveil the potential use of AgNPs in sensing, bioimaging, and other applications related to neurotransmitters. The optimized geometries of Ag_n_ clusters and dopamine are presented in Figure 1. Lee et al. reported the structures of pure silver clusters (Agn, *n* = 1–13) and neutral and anionic gold–silver binary clusters by using DFT with generalized gradient approximation and high-level ab initio calculations including coupled cluster theory with relativistic ab initio pseudopotentials.^49^ McKee and Samokhvalov^50^ investigated the geometries and electronic properties of neutral Ag_n_ clusters, with n ranging from 2 to 22, using DFT. Their study revealed that the transition from planar to “empty cage” structures occurs at *n* = 7. Subsequently, a transition from “empty cage” to “cage with one Ag atom” takes place at *n* = 18 and further to “cage with two Ag atoms” at *n* = 22. The geometries of the most stable Ag_n_ clusters discussed in this study are based on these findings.

**Figure 1:**
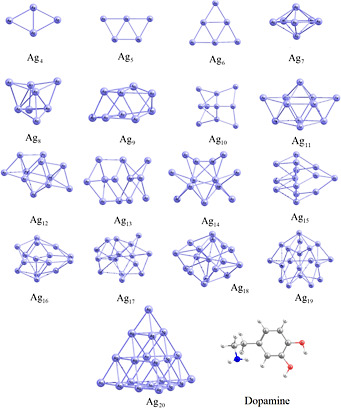
Relaxed geometries of the studied silver clusters and dopamine.

In order to investigate the adsorption of dopamine onto AgNPs, the geometry optimization of DOP@Ag_n_ nanohybrids is performed in different configurations. In the most stable configuration, the Ag atom of clusters interacts with the amine group of the ethylamine side chain of dopamine. The obtained configurations are considered for further analysis. The adsorption energies of DOP@Ag_n_ in the gas phase are listed in Table 1. The optimized geometries of the most favorable structures of DOP@Ag_n_ nanohybrids are depicted in Figure 2. The interaction between Ag_n_ clusters and the amine group of the ethylamine side chain in dopamine results in adsorption energy values ranging from −18.8 to −28.1 kcal/mol. The maximum adsorption energy occurs in the DOP@Ag_4_ complex, while the minimum is observed in the DOP@Ag_15_ complex. All nanohybrids exhibit significant adsorption energy, indicating strong chemisorption of dopamine onto silver nanoclusters. These energies demonstrate that Ag clusters interact intensively with the ethylamine side chain of the dopamine molecule.

**Table 1:** Calculated Adsorption Energy (*E*_ads_ in kcal/mol), Dipole Moment (μ in Debye), and Solvation Energy (*E*_sol_ in kcal/mol)

nanohybrid	*E*_ads_ (water phase)	*E*_ads_ (gas phase)	*E*_sol_ (kcal/mol)	μ (water phase)	μ (gas phase)
DOP@Ag_4_	–30.9	–28.1	–2.8	6.1	4.2
DOP@Ag_5_	–23.8	–20.8	–5.0	5.1	4.6
DOP@Ag_6_	–26.5	–21.0	–5.5	5.2	4.8
DOP@Ag_7_	–24.9	–20.3	–4.7	6.4	4.6
DOP@Ag_8_	–24.7	–20.0	–4.8	6.0	5.0
DOP@Ag_9_	–27.2	–22.0	–5.2	8.6	6.2
DOP@Ag_10_	–27.7	–21.6	–6.1	5.7	4.8
DOP@Ag_11_	–27.2	–22.5	–4.7	5.5	4.3
DOP@Ag_12_	–25.7	–21.0	–4.8	7.3	5.7
DOP@Ag_13_	–26.4	–21.0	–5.4	6.9	4.8
DOP@Ag_14_	–27.9	–23.1	–4.7	5.8	5.5
DOP@Ag_15_	–24.7	–20.0	–5.0	8.1	3.9
DOP@Ag_16_	–25.5	–25.2	–0.2	6.6	2.8
DOP@Ag_17_	–32.4	–23.9	–8.4	4.9	3.5
DOP@Ag_18_	–25.2	–20.7	–4.5	6.9	6.6
DOP@Ag_19_	–27.3	–21.1	–6.2	7.2	5.2
DOP@Ag_20_	–29.2	–21.6	–7.6	5.8	5.2

**Figure 2:**
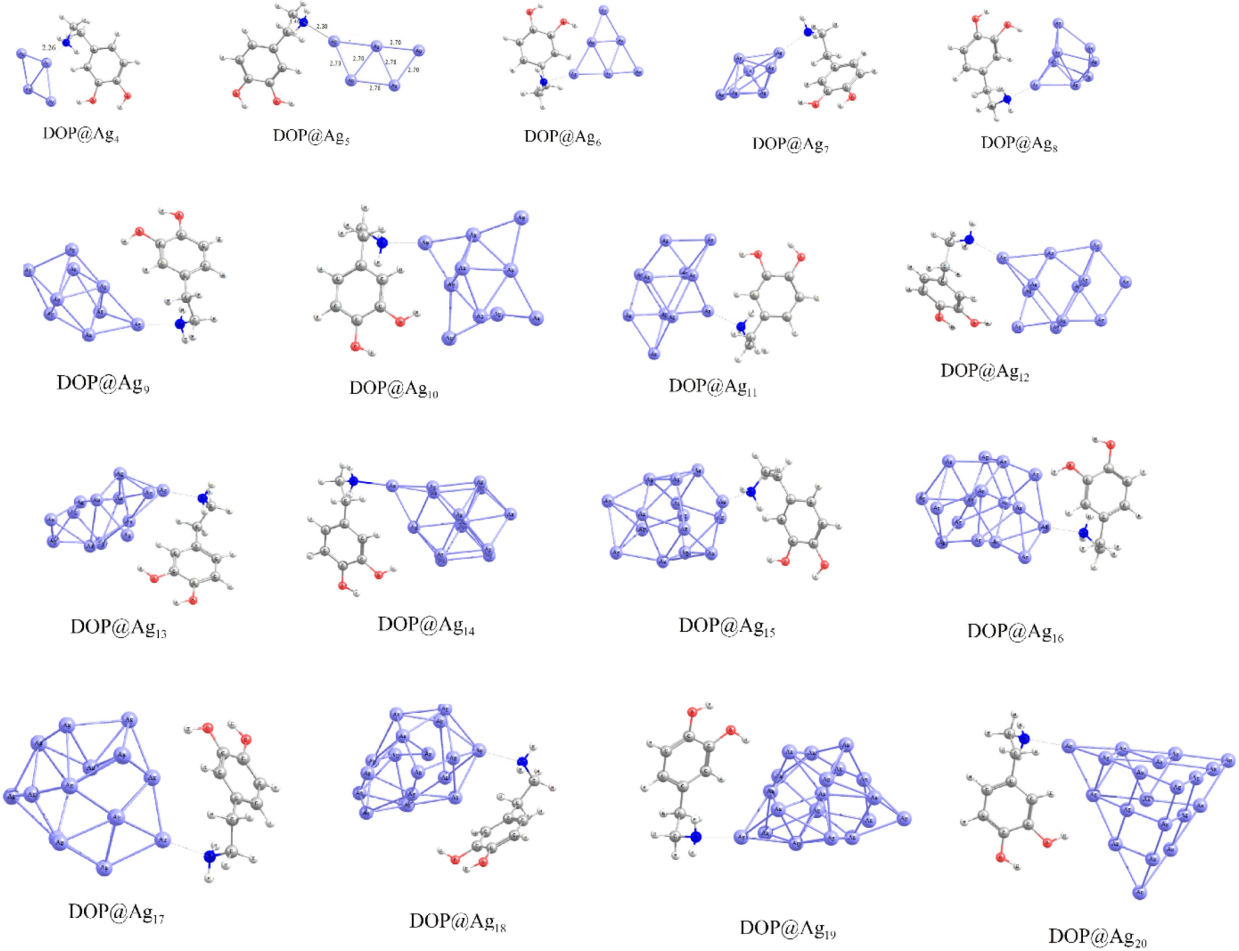
Most stable DOP@Ag_n_ nanohybrids.

In order to verify the model for the study, the DOP@Ag_4_ nanohybrid was investigated further. This nanohybrid, along with its isolated components (Ag_4_ and DOP), was optimized using several computational functionals, including BLYP, PBE0, B3LYP, PBE0-D3, and B3LYP-D3. The coupled-cluster CCSD­(T) method was employed as a reference standard for comparison. All calculations utilized a uniform basis set, specifically LANL2DZ with an ECP for silver atoms and 6–311++G** for the dopamine molecule. The adsorption energy for the DOP@Ag_4_ complex, as determined by using the CCSD­(T) method, was found to be −26.2 kcal/mol. The calculated adsorption energies derived from BLYP, B3LYP, and PBE0 functionals were significantly underestimated relative to the CCSD­(T) result, yielding values of −20.2, −20.3, and −23.8 kcal/mol, respectively. Notably, when dispersion corrections were included, the adsorption energies approached those obtained from the CCSD­(T) method. The values obtained from LC-BLYP, BLYP-D3, B3LYP-D3, CAM-B3LYP, and PBE0-D3 functionals were −28.1, −31.2, −29.6, −30.8, and −30.2 kcal/mol, respectively. The “D3” designation refers to the D3 version of Grimme’s dispersion model with the original D3 damping function, while the prefix “LC-” indicates the application of long-range corrections as proposed by Hirao and colleagues. While all dispersion-corrected functionals tended to overestimate the adsorption energies compared to the CCSD­(T) method, the results obtained using the LC-BLYP functional were notably more reliable, exhibiting a relative error percentage of only 7%. Consequently, the LC-BLYP method was selected for further studies involving additional complexes. The inclusion of long-range dispersion corrections is critical for accurately modeling these systems, and the reliability of the LC-BLYP method for noble metals has been supported by previous research. Further, to evaluate the impact of an extended basis set on the adsorption energy calculations, the adsorption energy for the DOP@Ag_4_ complex was calculated by using optimized structures derived from the Def2TZVP basis set in conjunction with the LC-BLYP method. The resulting adsorption energy was found to be −27.6 kcal/mol. Also, an extended mixed basis set of aug-cc-pVDZ-PP for silver atoms and aug-cc-pVDZ for dopamine is applied and the adsorption energy is found to be-28.5 kcal/mol. The adsorption energy obtained using LANL2DZ with ECP for silver and 6–311++G** for dopamine is −28.1 kcal/mol, which closely aligns with the results from the larger basis sets. This agreement demonstrates that the LANL2DZ with ECP effectively captures the electronic structure of silver, while 6–311++G** provides an accurate description of dopamine. Consequently, this combination offers a reliable and computationally efficient approach, balancing accuracy with resource demands for subsequent calculations.

Noticeably, the preferred geometries of small silver clusters change upon molecular binding. The coordination preferences established for pure clusters are altered in the presence of a dopamine molecule. To understand how electronic-structure modifications influence the structural stability, deformation energy analyses were performed. The deformation energy of silver clusters upon dopamine adsorption is evaluated using the following equation

2 $$E_{\mathrm{def}}=E_{{Ag}_{n}}^{Relaxed}-E_{{Ag}_{n}}$$

where $E_{{Ag}_{n}}^{Relaxed}$ is the total energy of the Ag_n_ cluster in its relaxed geometry on the DOP@Ag_n_ nanohybrid. This term is calculated via a single-point energy calculation of the Ag_n_ cluster in the optimized geometry of the nanohybrid. The *E*_Agn_ term also denotes the energy of the optimized isolated Ag_n_ cluster. The deformation energy represents the energy required for the silver clusters to adapt to an energetically favorable interaction with dopamine. The deformation energies are reported in Table 2 and range from 0.53 to 5.73 kcal/mol, with the maximum deformation observed for the Ag_10_ cluster. Additionally, to assess changes in structural arrangements, the geometry of each silver cluster in the isolated form is compared to its geometry within the corresponding DOP@Ag_n_ nanohybrid. Root-mean-square deviations (RMSDs) between the two structures are calculated, and the minimum interatomic distances between the corresponding atoms in the isolated and relaxed clusters are reported in Table 2. For each nanohybrid, the average RMSD is provided as well. Notably, the largest average RMSD corresponds to Ag_10_ (0.633), together with the greatest deformation energy (5.73 kcal/mol). The obtained average RMSDs exhibit a linear correlation with deformation energies, with a regression coefficient *R* ^2^ = 0.98.

**Table 2:** Root-Mean-Square Deviations (RMSDs) Between Isolated Ag_n_ and Relaxed Ag_n_ in Nanohybrids, the Minimum Interatomic Distances Between Corresponding Atoms in the Isolated and Relaxed Silver Clusters, and their Deformation Energy (*E*_def_)

atom	Ag_4_	Ag_5_	Ag_6_	Ag_7_	Ag_8_	Ag_9_	Ag_10_	Ag_11_	Ag_12_	Ag_13_	Ag_14_	Ag_15_	Ag_16_	Ag_17_	Ag_18_	Ag_19_	Ag_20_
RMSD-Ag1	0.07	0.336	0.066	0.046	0.059	0.022	0.098	0.105	0.029	0.045	0.044	0.101	0.059	0.386	0.077	0.113	0.017
RMSD-Ag2	0.051	0.042	0.229	0.028	0.044	0.039	0.388	0.029	0.11	0.016	0.067	0.124	0.189	0.697	0.049	0.017	0.004
RMSD-Ag3	0.078	0.31	0.171	0.056	0.025	0.058	1.585	0.047	0.076	0.071	0.068	0.118	0.1	0.243	0.011	0.031	0.005
RMSD-Ag4	0.05	0.267	0.03	0.062	0.057	0.043	0.999	0.057	0.014	0.036	0.081	0.097	0.054	0.359	0.036	0.055	0.025
RMSD-Ag5	-	0.268	0.271	0.089	0.044	0.009	0.822	0.36	0.079	0.056	0.092	0.056	0.024	0.487	0.064	0.024	0.007
RMSD-Ag6	-	-	0.306	0.022	0.086	0.043	0.881	0.04	0.038	0.059	0.021	0.084	0.138	0.492	0.063	0.023	0.019
RMSD-Ag7	-	-	-	0.05	0.028	0.053	0.338	0.035	0.025	0.073	0.01	0.017	0.053	0.422	0.058	0.024	0.017
RMSD-Ag8	-	-	-	-	0.048	0.072	0.3	0.035	0.036	0.053	0.009	0.137	0.076	0.357	0.037	0.034	0.015
RMSD-Ag9	-	-	-	-	-	0.063	0.572	0.018	0.015	0.029	0.052	0.028	0.011	0.109	0.058	0.016	0.007
RMSD-Ag10	-	-	-	-	-	-	0.345	0.02	0.084	0.02	0.011	0.141	0.031	0.471	0.031	0.02	0.008
RMSD-Ag11	-	-	-	-	-	-	-	0.031	0.035	0.095	0.035	0.057	0.025	0.105	0.021	0.017	0.017
RMSD-Ag12	-	-	-	-	-	-	-	-	0.039	0.056	0.026	0.044	0.046	0.431	0.025	0.021	0.017
RMSD-Ag13	-	-	-	-	-	-	-	-	-	0.083	0.023	0.112	0.019	0.181	0.059	0.03	0.06
RMSD-Ag14	-	-	-	-	-	-	-	-	-	-	0.021	0.081	0.046	0.838	0.042	0.025	0.014
RMSD-Ag15	-	-	-	-	-	-	-	-	-	-	-	0.015	0.06	0.416	0.013	0.072	0.054
RMSD-Ag16	-	-	-	-	-	-	-	-	-	-	-	-	0.051	0.797	0.046	0.032	0.091
RMSD-Ag17	-	-	-	-	-	-	-	-	-	-	-	-	-	0.901	0.031	0.023	0.04
RMSD-Ag18	-	-	-	-	-	-	-	-	-	-	-	-	-	-	0.037	0.031	0.052
RMSD-Ag19	-	-	-	-	-	-	-	-	-	-	-	-	-	-	-	0.051	0.017
RMSD-Ag20	-	-	-	-	-	-	-	-	-	-	-	-	-	-	-	-	0.026
Average RMSD	0.062	0.245	0.179	0.05	0.391	0.045	0.633	0.071	0.048	0.053	0.04	0.081	0.061	0.452	0.042	0.035	0.026
*E*_def_	0.528	1.862	1.417	0.818	0.693	0.657	5.737	0.675	0.613	0.79	0.857	0.65	0.69	3.87	0.757	0.687	0.625

The work function (Φ) for a semiconductor can be defined as the minimum quantity of energy needed to separate an electron from the Fermi level to a point far enough from the system to sense any efficacy. The variation of work function of an adsorbent sheet during the gas adsorption process changes its field emission characteristics. The output of work function variations upon gas adsorption can be monitored via suspended gate field effect machines. This technique has been adopted as an efficient approach for the detection of various gas molecules. eq 3 describes the current densities of the emitted electron in vacuum

3 $$j={AT}^{2}e^{\left(-\Phi /kT\right)}$$

In eq 3, *A* is the Richardson constant (*A*/m^2^), *T* is the temperature (K), *k* is the Boltzmann constant, and Φ (eV) is the work function. The Φ values can be calculated using eq 4

4 $$\Phi =E_{\infty }-E_{F}=-\frac{E_{HOMO}+E_{LUMO}}{2}$$

In eq 3, *E*_∞_ is the electrostatic potential at infinity, which can be considered as zero and *E*_F_ is the Fermi level energy. It can be said that the Fermi level in a molecule at *T* = 0 K is located nearly in the middle of the HOMO–LUMO gap. Accordingly, the change in field emission (*j*) during dopamine adsorption at room temperature can be considered as

5 $$\frac{j_{DOP@Ag_{n}}}{j_{Ag_{n}}}=\exp\left(\frac{\Phi_{Ag_{n}}-\Phi_{DOP@Ag_{n}}}{kT}\right)$$

The calculated highest occupied molecular orbital (HOMO), lowest unoccupied molecular orbital (LUMO) energies, and work functions for isolated silver clusters and DOP@Ag_n_ nanohybrids are summarized in Table 3. Results indicate that dopamine adsorption onto Ag_n_ clusters reduces the work function by approximately 3–11%. The maximum decrease in the work function upon dopamine adsorption is observed in Ag_9_, with a reduction of 11%. Based on the results presented in Table 3, dopamine adsorption onto silver clusters leads to destabilization of both the HOMO and LUMO energy levels. However, the destabilization of the LUMO level is more pronounced than that of the HOMO level. If both energy levels were destabilized by the same magnitude, then the work function would remain unchanged. For instance, in the case of DOP@Ag_9_, the LUMO level experiences a relative destabilization of 26%, whereas the HOMO is destabilized by only 7%. This differential destabilization causes a decrease in the work function following dopamine adsorption onto the Ag_9_ cluster. A similar trend is observed across other silver clusters as well. Additionally, the ratios of field emission upon dopamine adsorption onto silver clusters were calculated, revealing a substantial increase. These ratios range from 70 for Ag_11_ and Ag_20_ clusters to 9 × 10^6^ for the Ag_6_ cluster. Notably, these results suggest that the studied silver clusters could serve as promising work function-based sensors for dopamine detection. Three-dimensional maps of the HOMO–LUMO electron distribution of the DOP@Ag_n_ nanohybrids are presented in Figure 3. After adsorption of dopamine on Ag clusters, the LUMO remained unchanged, keeping a uniform distribution across the cluster, whereas the HOMO slightly concentrated on the dopamine molecule, with a portion delocalized amidst the cluster and dopamine molecule. The observed overlap between the dopamine molecule and the silver clusters indicates strong interactions.

**Table 3:** Calculated HOMO (*E*_HOMO_) and LUMO (*E*_LUMO_) Energies Together with Work Function (Φ) and Its Relative Change (%ΔΦ) Due to Dopamine Adsorption onto Silver Clusters. The ratio of field emission upon dopamine adsorption is provided in the last column

cluster	*E*_HOMO_	*E*_LUMO_	Φ	nanohybrid	*E*_HOMO_	*E*_LUMO_	Φ	%ΔΦ	$\frac{j_{DOP@{Ag}_{n}}}{j_{Ag_{n}}}$
Ag_4_	–6.27	–1.01	3.64	DOP@Ag_4_	–6.24	–0.31	3.27	–10	2 × 10^6^
Ag_5_	–6.15	–1.27	3.71	DOP@Ag_5_	–5.81	–0.90	3.36	–9	8 × 10^5^
Ag_6_	–7.18	–0.64	3.91	DOP@Ag_6_	–6.69	–0.37	3.53	–10	3 × 10^6^
Ag_7_	–5.74	–1.08	3.41	DOP@Ag_7_	–5.55	–0.92	3.23	–5	1 × 10^3^
Ag_8_	–6.98	–0.51	3.74	DOP@Ag_8_	–6.60	–0.36	3.48	–7	3 × 10^4^
Ag_9_	–5.99	–1.61	3.80	DOP@Ag_9_	–5.59	–1.19	3.39	–11	9 × 10^6^
Ag_10_	–6.31	–1.14	3.73	DOP@Ag_10_	–5.92	–0.86	3.39	–9	6 × 10^5^
Ag_11_	–5.49	–1.35	3.42	DOP@Ag_11_	–5.43	–1.19	3.31	–5	7 × 10^1^
Ag_12_	–6.07	–1.37	3.72	DOP@Ag_12_	–5.87	–1.12	3.50	–6	5 × 10^3^
Ag_13_	–5.46	–1.54	3.50	DOP@Ag_13_	–5.31	–1.33	3.32	–5	1 × 10^3^
Ag_14_	–5.67	–1.25	3.46	DOP@Ag_14_	–5.47	–1.12	3.30	–5	5 × 10^2^
Ag_15_	–5.47	–1.54	3.51	DOP@Ag_15_	–5.32	–1.28	3.30	–6	4 × 10^3^
Ag_16_	–5.72	–1.66	3.69	DOP@Ag_16_	–5.60	–1.56	3.58	–3	7 × 10^1^
Ag_17_	–5.86	–1.93	3.90	DOP@Ag_17_	–5.32	–1.72	3.52	–10	3 × 10^6^
Ag_18_	–5.63	–1.33	3.48	DOP@Ag_18_	–5.33	–0.99	3.16	–9	3 × 10^5^
Ag_19_	–5.15	–1.33	3.24	DOP@Ag_19_	–5.01	–1.20	3.11	–4	2 × 10^2^
Ag_20_	–6.54	–1.01	3.78	DOP@Ag_20_	–6.41	–0.93	3.67	–3	7 × 10^1^

**Figure 3:**
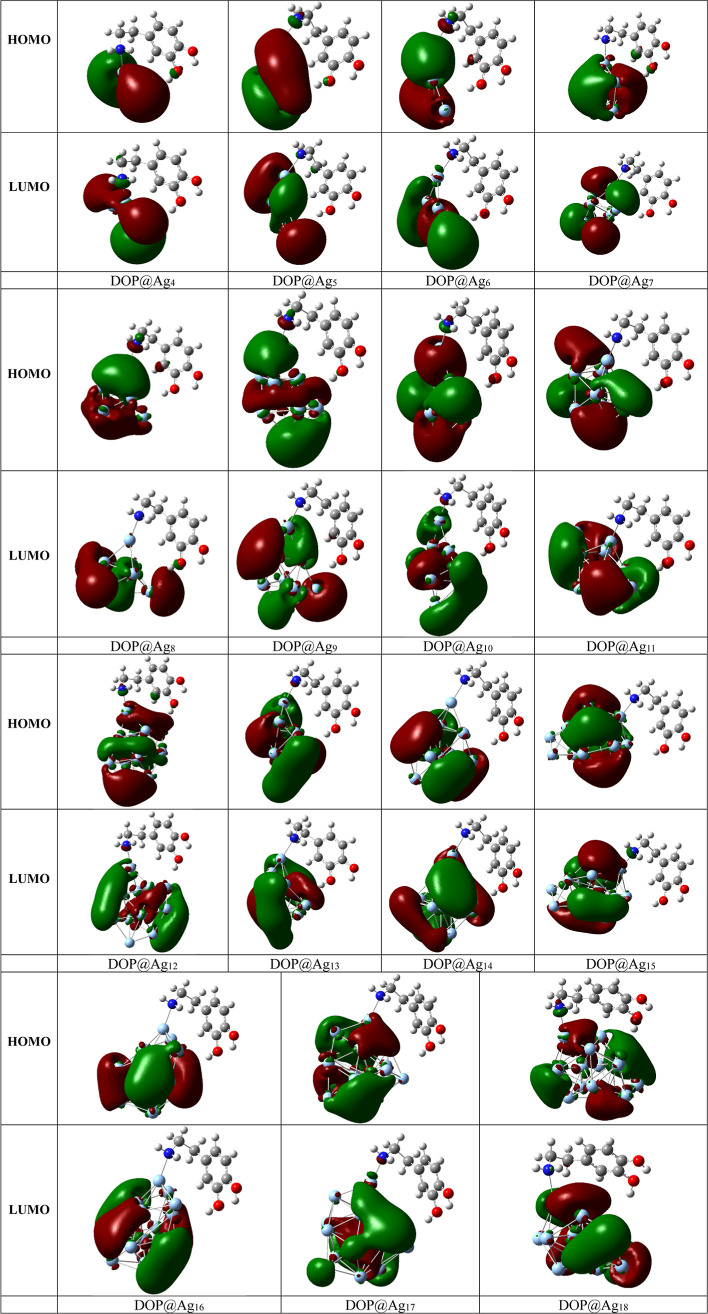

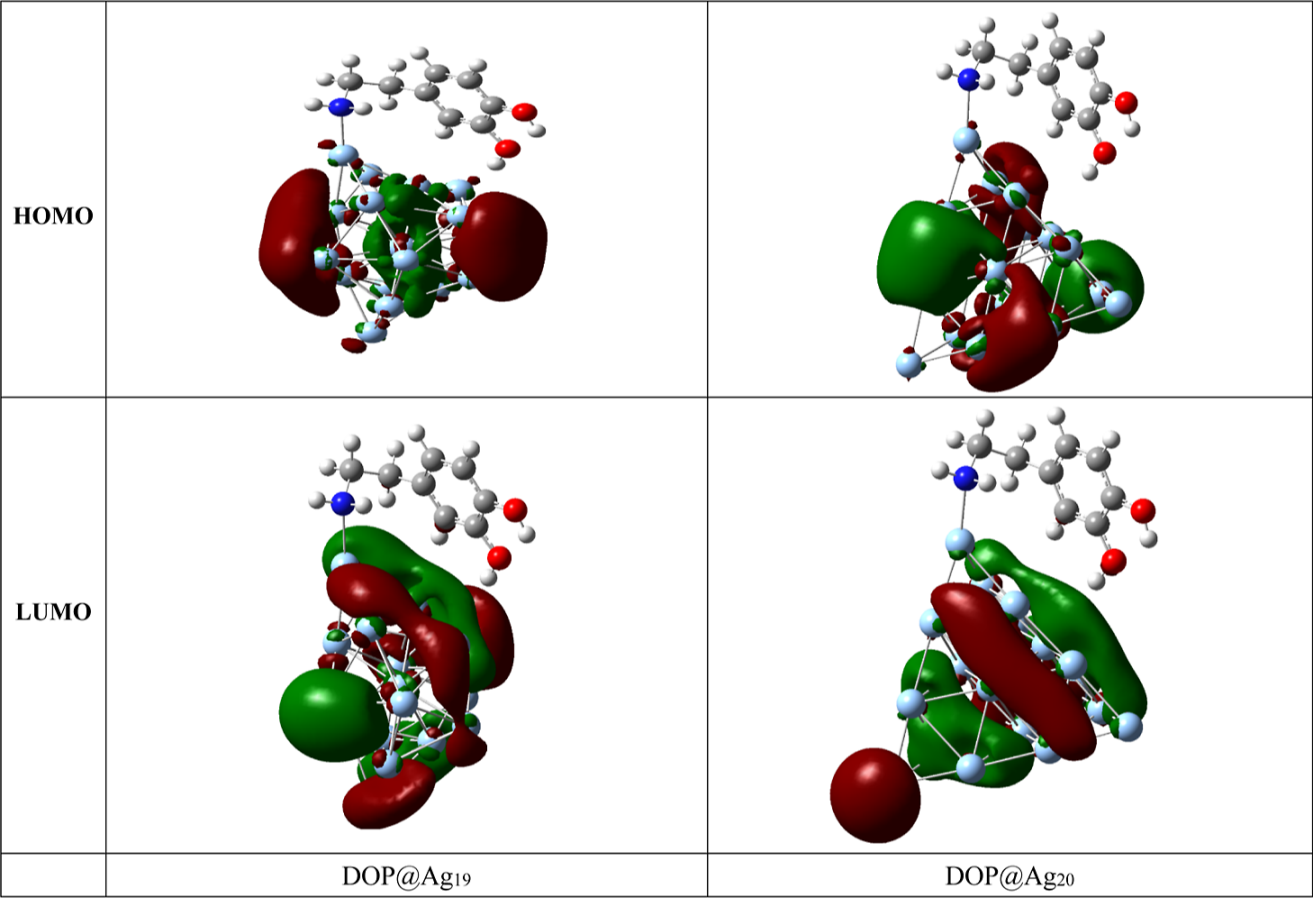
Three-dimensional maps of the HOMO–LUMO electron distribution of the DOP@Ag_n_ nanohybrids.

Molecular electrostatic potential (MEP) plays a determining role in elucidating interactions between nanostructured materials and biomolecules by revealing charge density distribution within biomolecular systems. This insight is very crucial in the prediction and enhancement of binding sites with more accuracy. By utilization of MEP, we can explore spots on biomolecules that are prone to interact with particular regions on nanomaterials, leading to the development of more effective nanomaterial-based systems for their active employment in bioimaging, drug delivery, and sensing applications. In MEP maps, colors usually reveal regions with charge densities. Red and yellow often denote areas with negative electrostatic potential, which are abundant in electrons and may serve as targets for interactions with positively charged species. Positive electrostatic potential areas are shown in blue; these are electron-deficient regions that are likely to interact with negatively charged species. Areas of negative electrostatic potential are usually indicted by green colors. Figure 4 shows the MEP surfaces of isolated monomers and nanohybrids of DOP@Ag_n_, respectively. The results of this figure indicate that the interaction occurs between the silver atom in the Ag_n_ nanoclusters, characterized by electron deficiency shown in the blue MEP region, and the nitrogen atom of the dopamine molecule, which exhibits a yellow MEP, indicating a relatively electron-rich site. As can be seen in nanohybrids, the regions near the hydrogen atoms of the NH_2_ groups of the ethylamine side chain are more positive in nanohybrids, making them favorable for the nucleophilic substitution reactions. The uniform distribution of MEP across both the cluster and dopamine molecules confirms the interaction between these fragments and the formation of the DOP@Ag_n_ nanohybrid.

**Figure 4:**
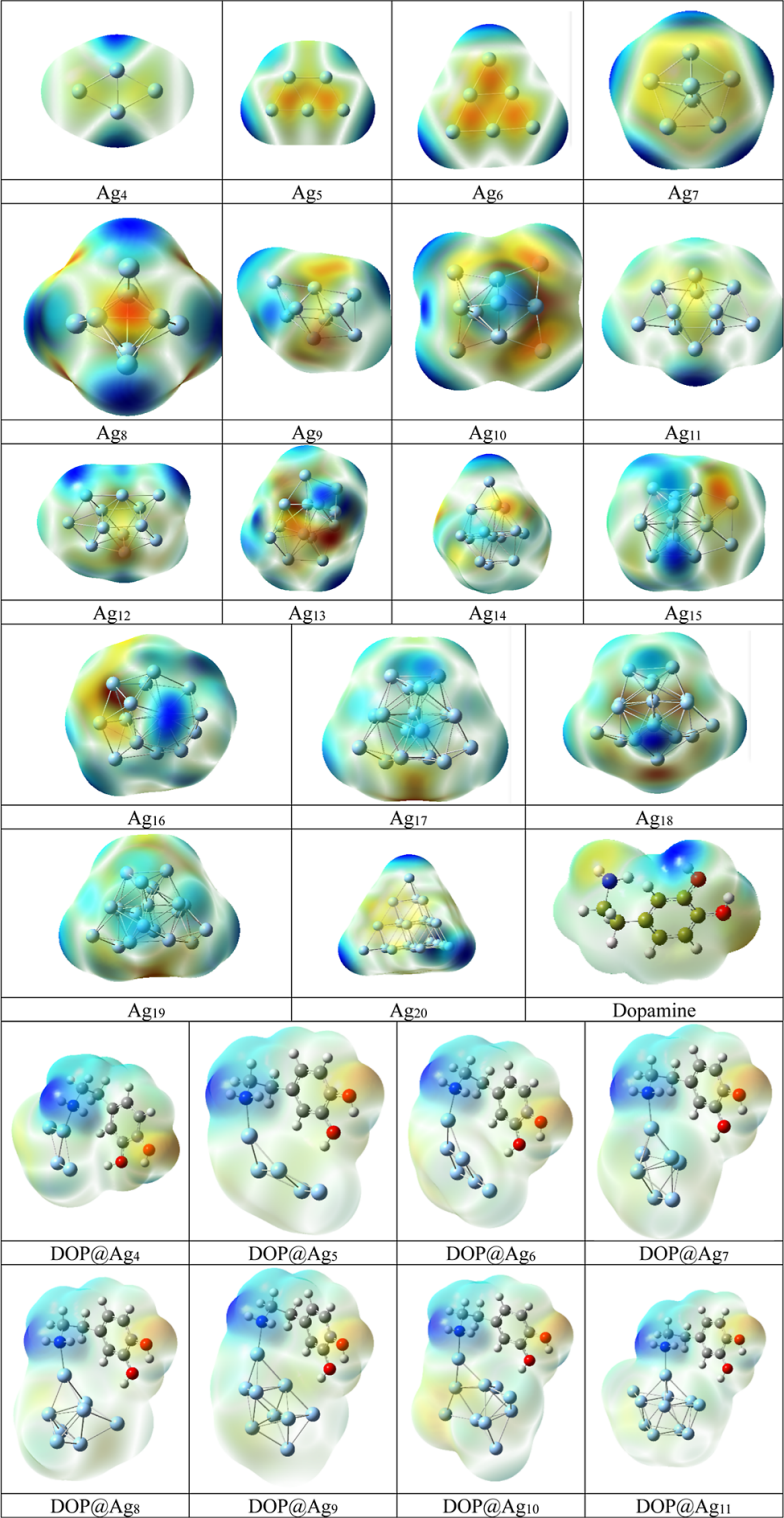

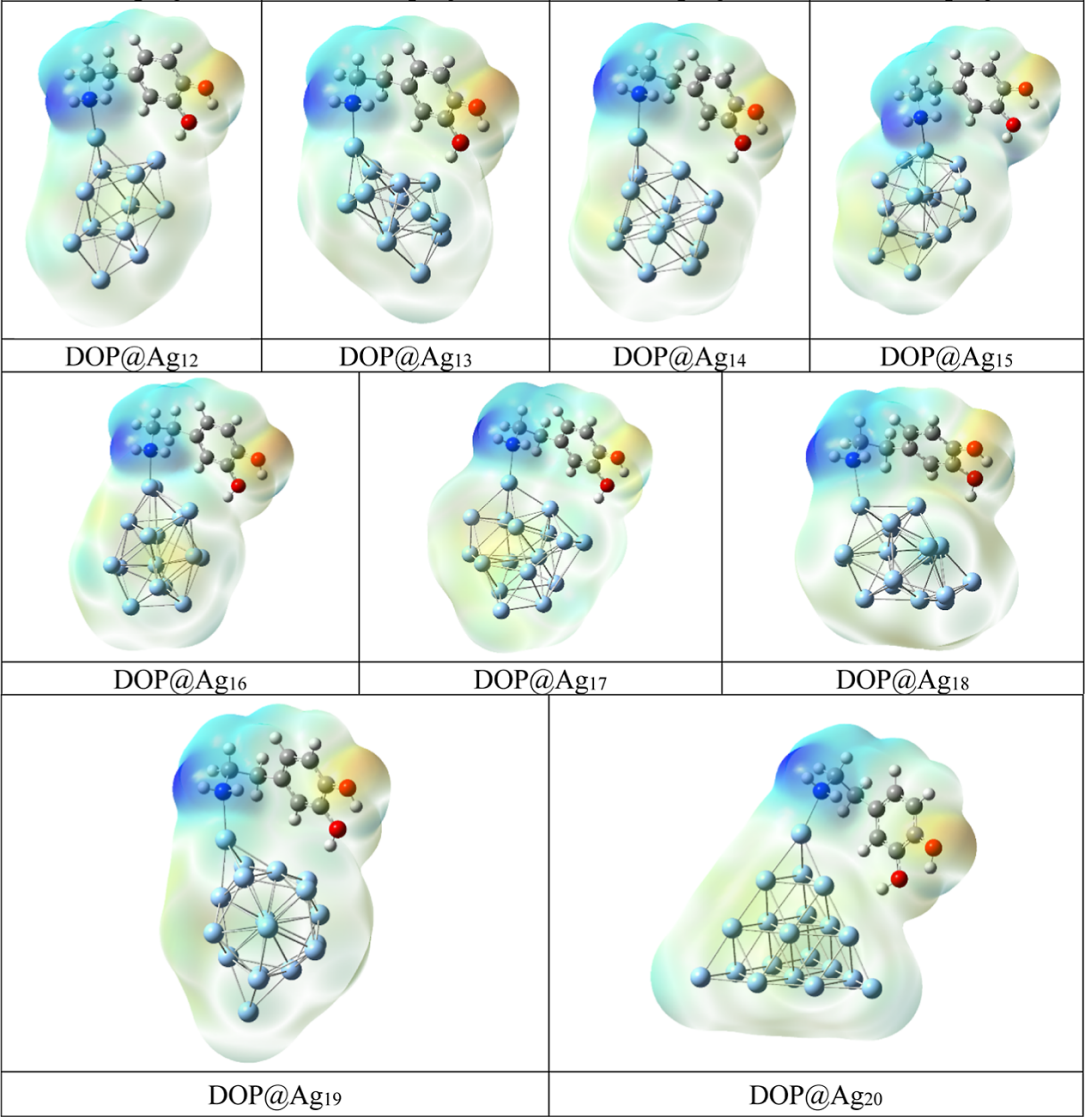
MEP surfaces of the studied structures.

The quantum theory of atoms in molecules (QTAIM) has proven to be an invaluable framework for elucidating the nature of chemical interactions through the analysis of electron density distributions.^51^ In QTAIM, the bond critical point (BCP) is defined as a point along a trajectory of maximum electron density connecting two atoms. Key topological parameters evaluated at the BCP include electron density (ρ), its Laplacian (∇^2^ρ), the total electron energy density (*H*), and its components: kinetic energy density (*G*) and potential energy density (*V*). These parameters facilitate the characterization of bonding interactions based on local indicators of electron and energy densities calculated at the BCPs. Negative and positive Laplacian values at the BCP are indicative of “electron-shared” and electron-closed-shell interactions, respectively. This categorization is further refined by Bianchi et al.^52^ into three bonding regimes based on the absolute ratio of the potential energy density to the kinetic energy density. The QTAIM analysis of the most stable configurations is performed using the Multiwfn code.^53,54^ In all examined systems, a singular BCP is identified between the nitrogen atom of dopamine and a silver atom within the Ag_n_ cluster, suggesting a chemical interaction in the analyzed nanohybrids. The local topological parameters associated with this BCP of each configuration are summarized in Table 4. According to the data presented and the classification established by Bianchi et al.,^52^ the observed $\left|V_{BCP}\right|G_{BCP}$ ratios (ranging from 1.11 to 1.15) fall within the intermediate bonding regime, situated between electron-shared covalent bonds (with ratios greater than 2) and closed-shell ionic bonds and van der Waals interactions (with ratios lower than 1), which include dative bonds and ionic bonds of a weak covalent character. Furthermore, the low electron density values, significant positive Laplacian values, and small negative energy densities associated with these Ag–N bonds support the assertion of a dative bond exhibiting a strong ionic character.

**Table 4:** Topology Parameters for Bond Critical Point of Ag–N in Studied DOP@Ag_n_ Nanohybrids

BCP(Ag–N)	ρ	∇^2^ρ	*G*	*V*	*H*	$\left|V_{BCP}\right|/G_{BCP}$
DOP@Ag_4_	0.0658	0.2754	0.0807	–0.0925	–0.0118	1.15
DOP@Ag_5_	0.0611	0.2541	0.0735	–0.0834	–0.0100	1.13
DOP@Ag_6_	0.0598	0.2488	0.0716	–0.0810	–0.0094	1.13
DOP@Ag_7_	0.0575	0.2390	0.0682	–0.0767	–0.0085	1.12
DOP@Ag_8_	0.0598	0.2488	0.0716	–0.0810	–0.0094	1.13
DOP@Ag_9_	0.0606	0.2512	0.0725	–0.0822	–0.0097	1.13
DOP@Ag_10_	0.0619	0.2571	0.0745	–0.0848	–0.0103	1.14
DOP@Ag_11_	0.0612	0.2547	0.0736	–0.0836	–0.0099	1.14
DOP@Ag_12_	0.0595	0.2471	0.0710	–0.0803	–0.0093	1.13
DOP@Ag_13_	0.0571	0.2364	0.0674	–0.0757	–0.0083	1.12
DOP@Ag_14_	0.0595	0.2470	0.0710	–0.0803	–0.0093	1.13
DOP@Ag_15_	0.0613	0.2552	0.0738	–0.0837	–0.0099	1.13
DOP@Ag_16_	0.0557	0.2312	0.0656	–0.0734	–0.0078	1.12
DOP@Ag_17_	0.0607	0.2530	0.0730	–0.0827	–0.0098	1.13
DOP@Ag_18_	0.0513	0.2121	0.0592	–0.0653	–0.0062	1.10
DOP@Ag_19_	0.0582	0.2416	0.0691	–0.0779	–0.0087	1.13
DOP@Ag_20_	0.0589	0.2439	0.0700	–0.0791	–0.0091	1.13

Additionally, the reduced density gradient (RDG) analysis is utilized to characterize the nature of noncovalent interactions.^55^ The noncovalent interaction reduced-density gradient (NCI-RDG) method is based on the electron density (ρ) and the RDG­(∇ρ), which can be expressed in the following equation

6 $$s=\frac{1}{2{\left(3\pi^{2}\right)}^{1\backslash 3}}\frac{\left|\nabla \rho \right|}{\rho^{4\backslash 3}}$$

The NCI–RDG plots are generated using the Multiwfn program. The plots for the configurations under consideration are illustrated in Figure 5. Weak van der Waals interactions are represented by green disks at an isosurface value of 0.5 au The results clearly demonstrate the presence of weak intermolecular interactions between the neurotransmitters and Ag_n_.

**Figure 5:**
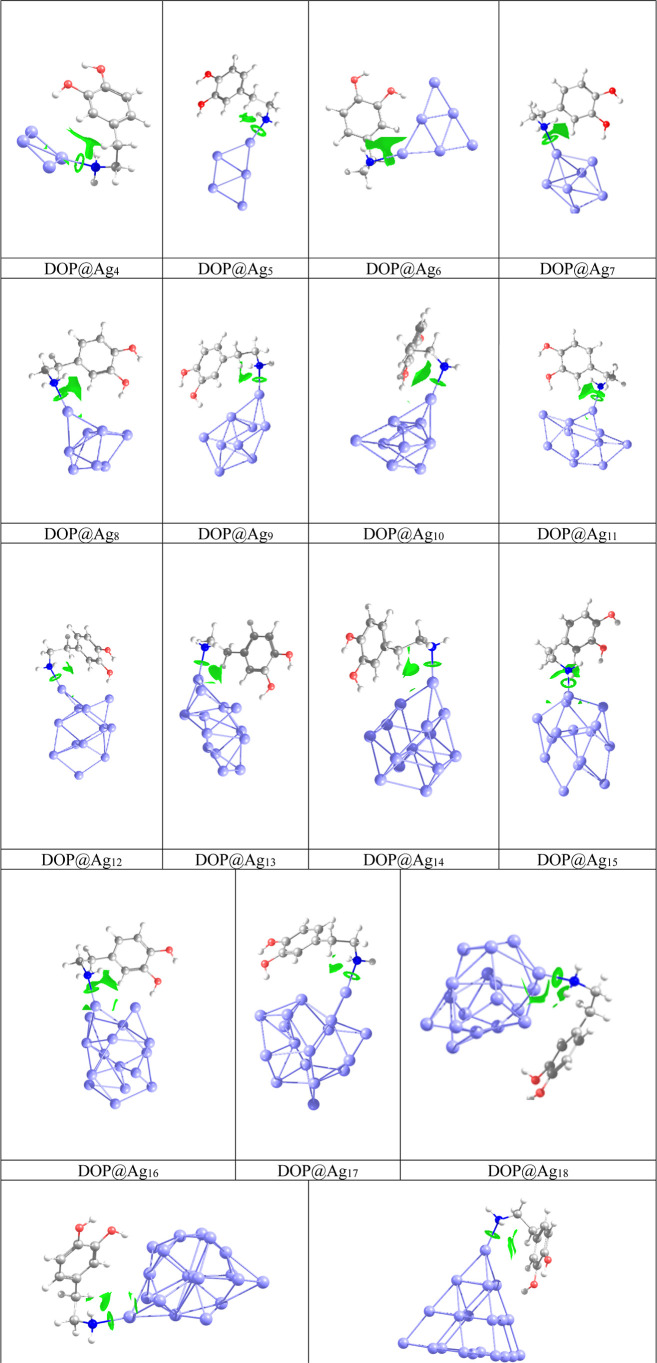
NCI–RDG plots for the DOP@Ag_n_ nanohybrids at RDG = 0.5.

The effect of the aqueous medium on the electronic properties and stability of DOP@Ag_n_ nanohybrids was investigated by using the IEF–PCM approach. Solvation energies were calculated to evaluate the stability of these complexes in water, as defined by

7 $$E_{sol}=E_{water}-E_{gas}$$

where *E*_sol_ represents the solvation energy, and *E*_water_ and *E*_gas_ denote the energies of studied DOP@Ag_n_ nanohybrids in water and gas phases, respectively. Table 1 reports the stability of DOP@Ag_n_ nanohybrids in water, indicated by favorable adsorption energies ranging from −32.4 to −23.8 kcal/mol. The negative solvation energies further confirm their stability in aqueous conditions. Notably, the DOP@Ag_17_ nanohybrid exhibited the highest adsorption energy of −32.4 kcal/mol, suggesting a strong interaction with water. Additionally, the dipole moments of the most stable configurations are summarized in Table 1. Results show a significant increase in dipole moments in the aqueous phase, implying improved solubility in polar solvents. This enhancement in the dipole moment correlates with increased electrostatic interactions, which are critical for water solubility, reactivity, and potential applications in targeted drug delivery. The increased adsorption energies and dipole moments in water indicate that the aqueous environment strengthens the interaction between silver clusters and dopamine, potentially improving their biological functions. Elevated dipole moments influence molecular interactions, binding affinity, and detection sensitivity—highlighting their importance in drug delivery and biosensing applications.

Thermal effects at physiological temperatures could influence the stability and interaction patterns. To further validate the thermal stability of our nanohybrid systems, we selected DOP@Ag_5_ and DOP@Ag_10_ as model systems at two sizes—Ag_5_ with an odd number of silver atoms and Ag_10_ with an even number— and performed ab initio molecular dynamics (AIMD) simulations using the DMol^3^ module.^56,57^ The computations for the simulation have been executed in a box of 25 × 25 × 25 Å with an *NVT* ensemble at a temperature value of 320 K. The *N*, *V*, and *T* terms stand for the number of atoms, volume, and temperature, respectively. The fluctuations in the total energy over dynamic steps is depicted in Figure 6. For a total of 3000 steps, a time step of 1 fs has been used. Besides, a profound generalized Gaussian moments thermostat has been included in the simulations. Figure 6 unveils the thermal stability of the most favorable nanohybrids. The total energy fluctuation that is considered as a standard criterion for the thermal stability has been found to be lower than 0.004 Ha for the considered nanohybrid system, and there was no significant deformation in structure during simulations. Accordingly, the considered nanohybrids are dynamically and structurally stable under the imposed circumstances, i.e., the structure of the considered nanohybrid remains unaltered through the simulations rendering its thermal stability. The above discussion highlights that the nanohybrid maintains robust thermal stability at 320 K.

**Figure 6:**
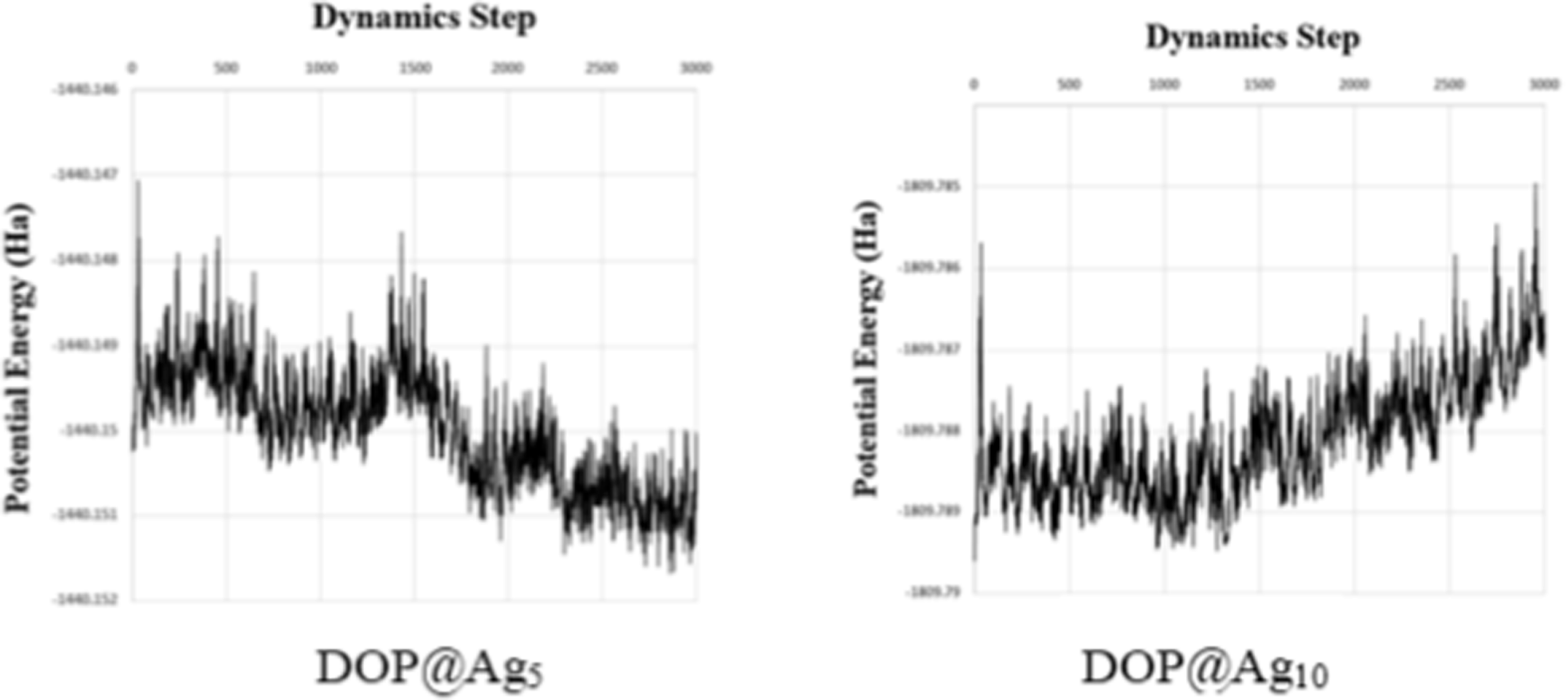
Alterations of the total energy (*NVT*) vs dynamic steps in the MD simulation at 320 K for the studied model complexes.

## Conclusion

In conclusion, the DFT comprehensive investigation into the interactions between dopamine neurotransmitter with silver clusters (Ag_n_ with *n* = 4–20) reveals significant insights into the potential applications of silver nanoparticles in biomedical applications, highlighting their promise for biosensing applications. The results reveal strong binding at the amine group of dopamine’s ethylamine side chain, with adsorption energies ranging from −20.8 to −28. 1 kcal/mol, indicating favorable interactions. The dopamine adsorption notably influences the electronic properties of silver nanoclusters, reducing the work function by up to 11% and substantially enhancing field emission ratios, particularly in Ag_9_. These modifications suggest that such nanohybrids could effectively alter field emission characteristics, a key feature for their use as work function-based sensors in drug detection. QTAIM analysis confirmed the formation of ionic dative Ag–N bonds in the studied nanohybrids, supporting the nature of the interactions. Furthermore, the study of DOP@Ag_n_ nanohybrids in aqueous environments indicates favorable adsorption energies between −23.8 and −32.4 kcal/mol, with negative solvation energies affirming their stability in water. The aqueous phase also exhibits a significant increase in dipole moments, implying improved solubility in polar solvents, which could enhance their reactivity and expand their applicability in targeted drug delivery. AIMD simulations verify the thermal and structural stability of selected nanohybrids at 320 K. Overall, these findings deepen our understanding of dopamine–silver nanocluster interactions and highlight their potential in developing effective biosensors. While further experimental validation is essential, these theoretical insights provide a valuable foundation that could streamline research efforts, reducing costs and time for practical implementation.

## Supplementary Material



## Notes

The corresponding vibrational spectra of the studied structures (Figure S1) (PDF)
