# Supplementary material for: Exploring Dopamine Neurotransmitter and Silver Nanocluster (Agn; n = 4–20) Interactions: DFT Insights for Biomedical Applications
Source: ACS Omega. 2025 Nov 25;10(48):58250–63. doi: 10.1021/acsomega.5c04404 (PMC12771186; doi:10.1021/acsomega.5c04404)
Supplement: Supplementary file 1 [file ao5c04404_si_001.pdf]

Electronic Supprting Information

Exploring Dopamine neurotransmitter and Silver Nanoclusters (Ag_n; n=4-20) Interactions: DFT Insights for Biomedical Applications

Ehsan Shakerzadeh[1], Elham Tahmasebi[1], Tarun Yadav[2], Noe Brigido Salvador[3], Ernesto Chigo Anota[*,3]

[1] *Chemistry Department, Faculty of Science, Shahid Chamran University of Ahvaz, Ahvaz, Iran*

[2] *School of Advanced Engineering, University of Petroleum and Energy Studies, Dehradun-248007, India*

[3]*Benemérita Universidad Autónoma de Puebla, Facultad de Ingeniería Química, Ciudad Universitaria, San Manuel, Puebla, Código Postal 72570, México*

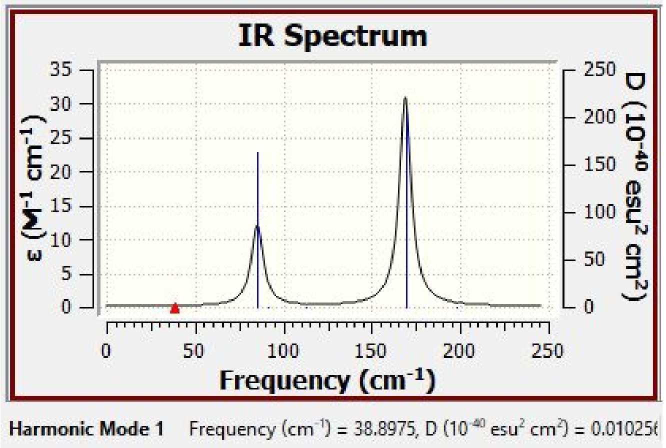

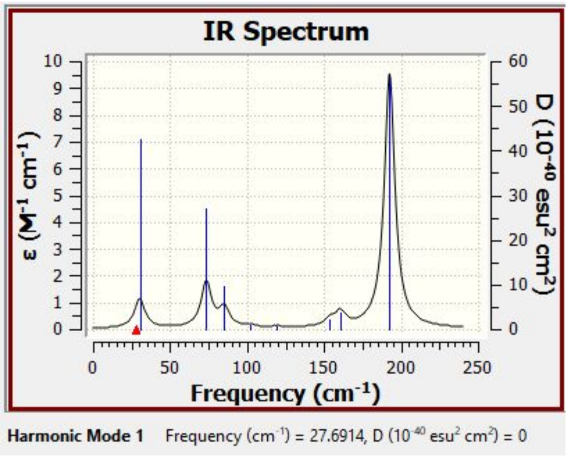

Ag_4 Ag_5

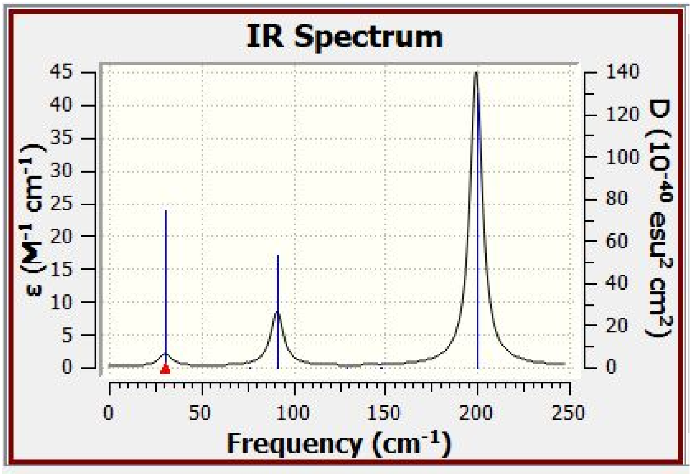

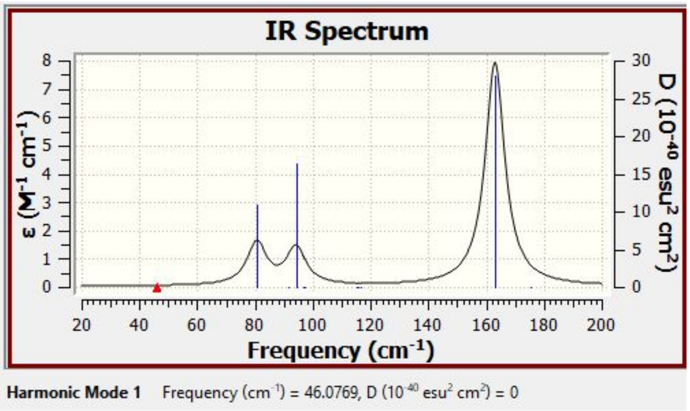

Ag_6 Ag_7

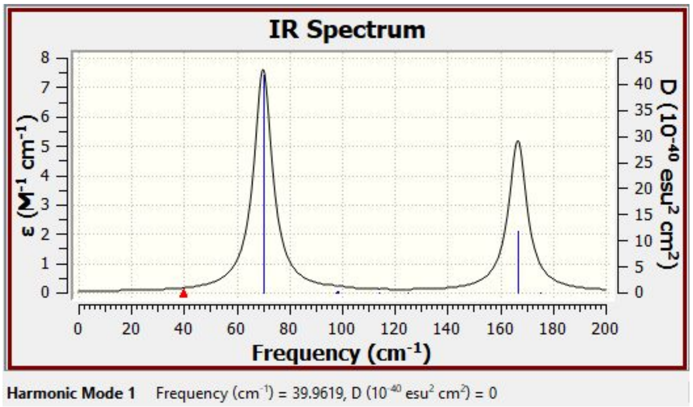

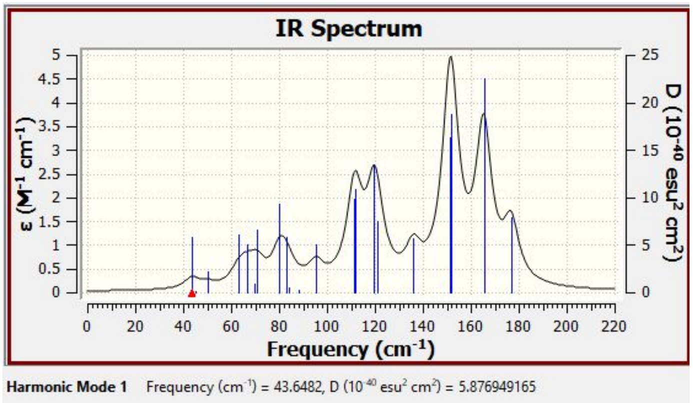

Ag_8 Ag_9

Fig. S1. The corresponding vibrational spectra.

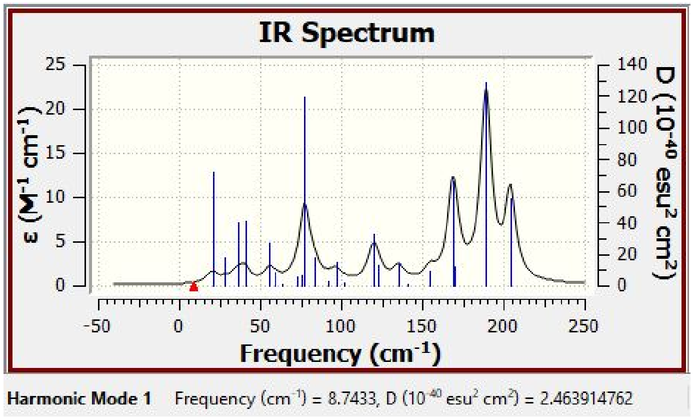

Ag10

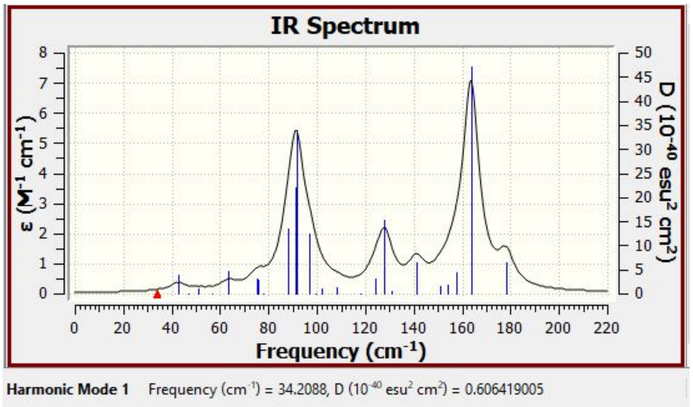

Ag11

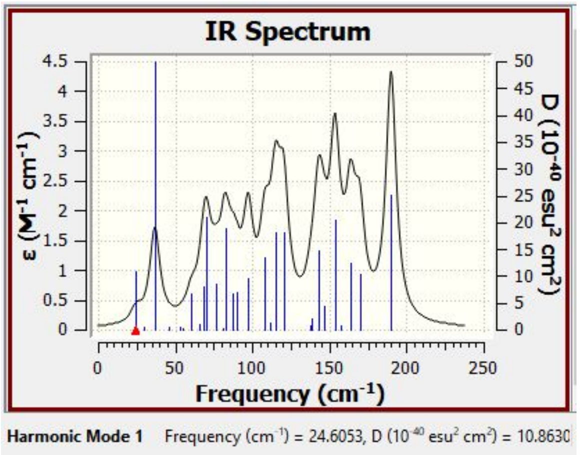

Ag12

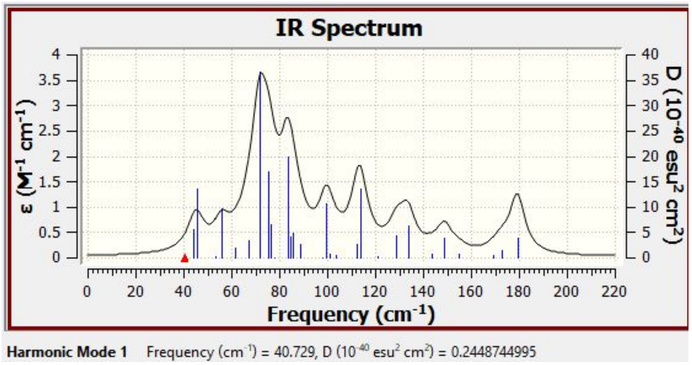

Ag13

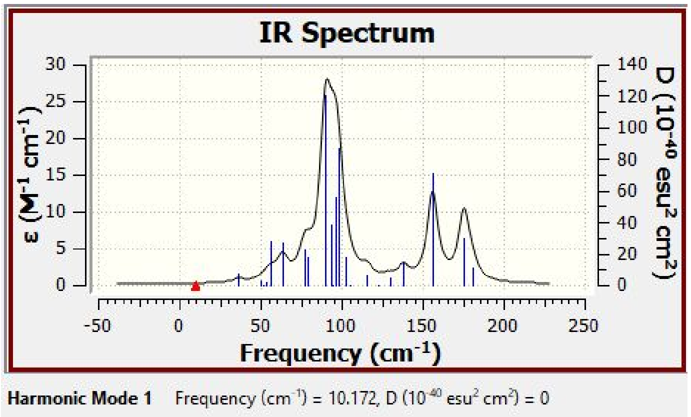

Ag14

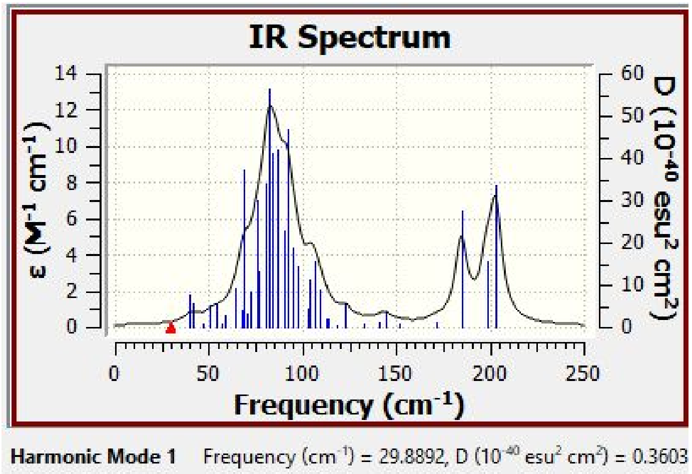

Ag15

Fig. S1. *Continued.*

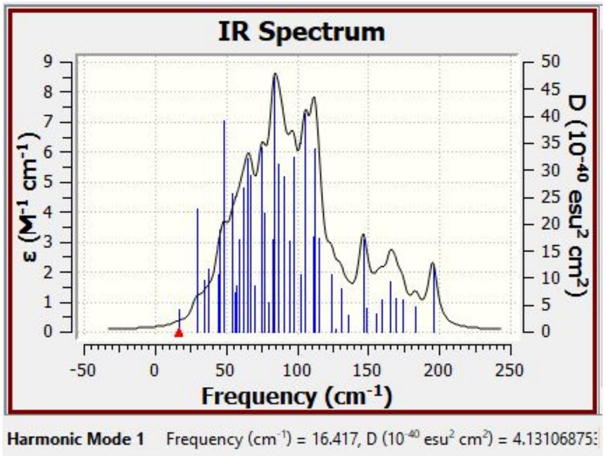

Ag$_{16}$

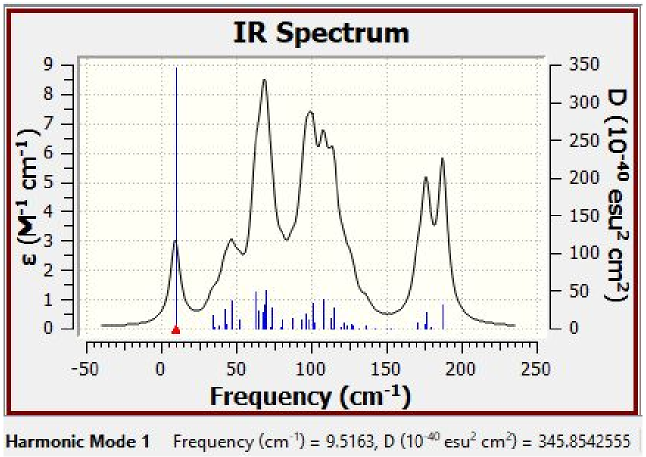

Ag$_{17}$

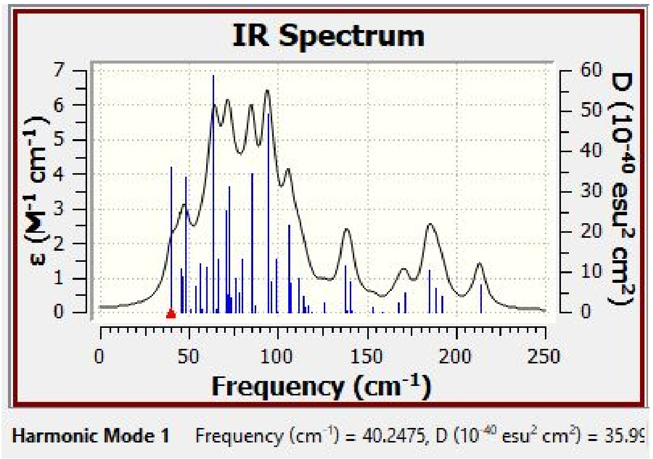

Ag$_{18}$

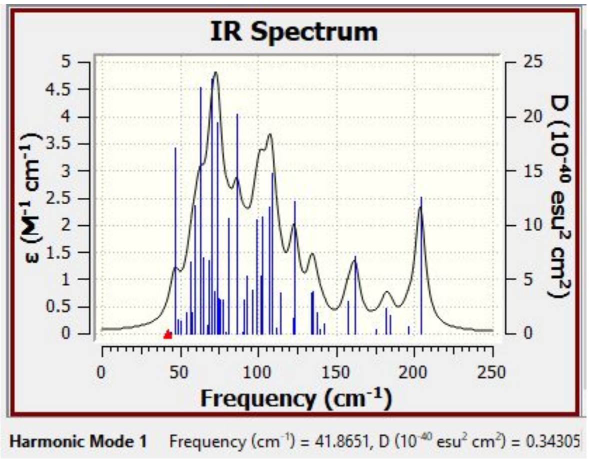

Ag$_{19}$

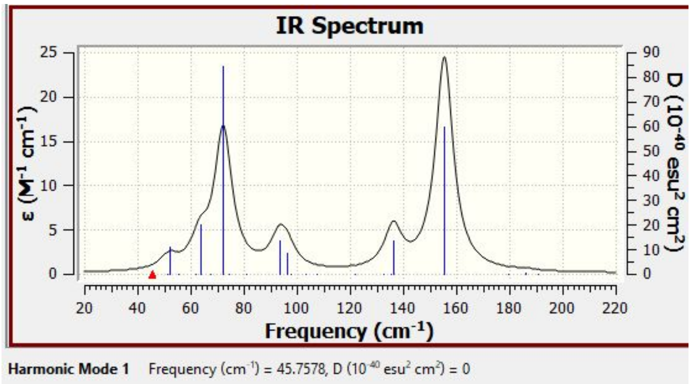

Ag$_{20}$

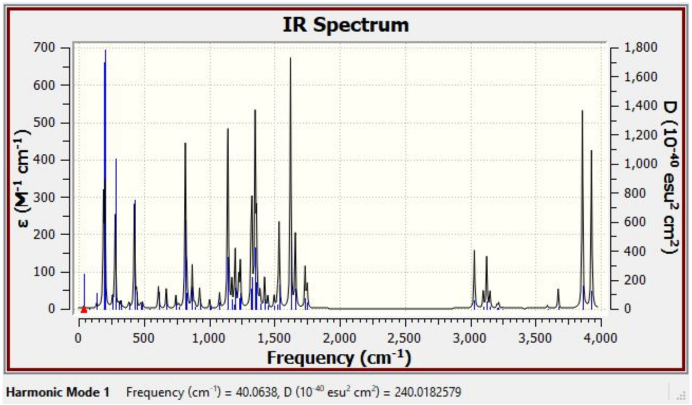

Dopamine

Fig. S1. *Continued.*

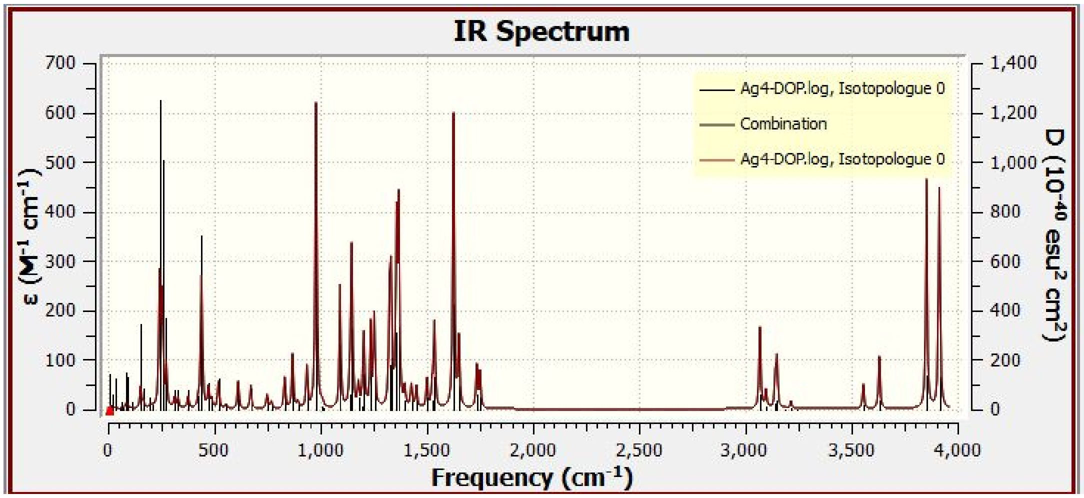

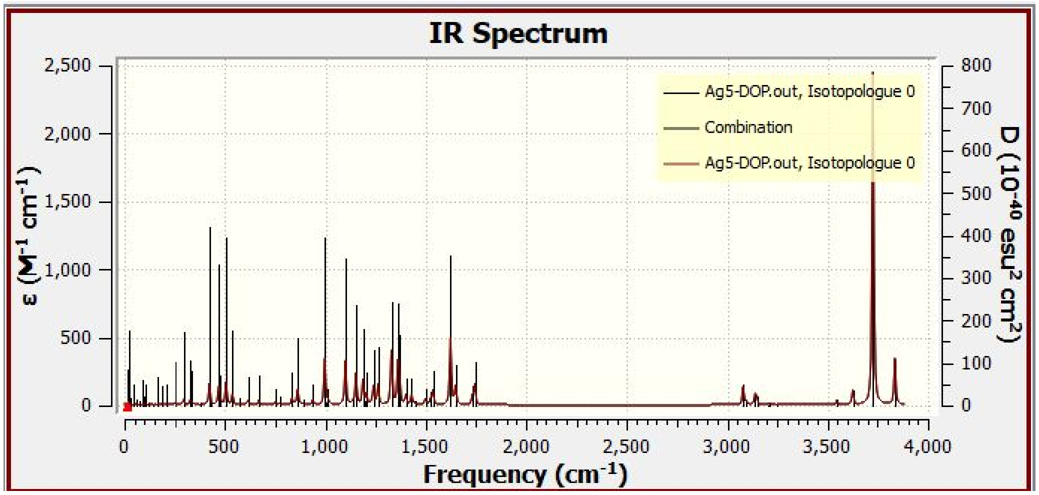

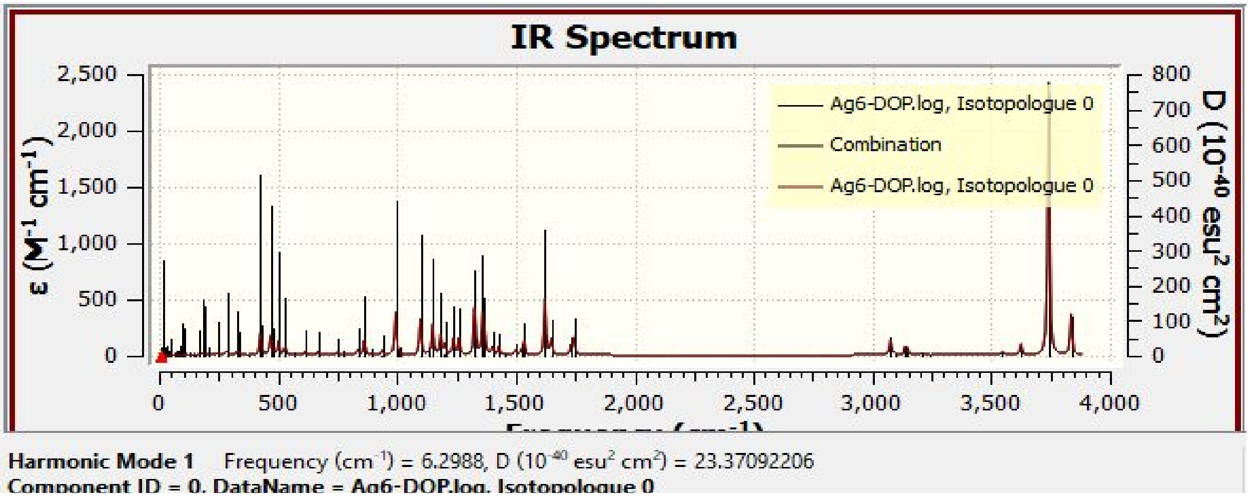

Fig. S1. *Continued.*

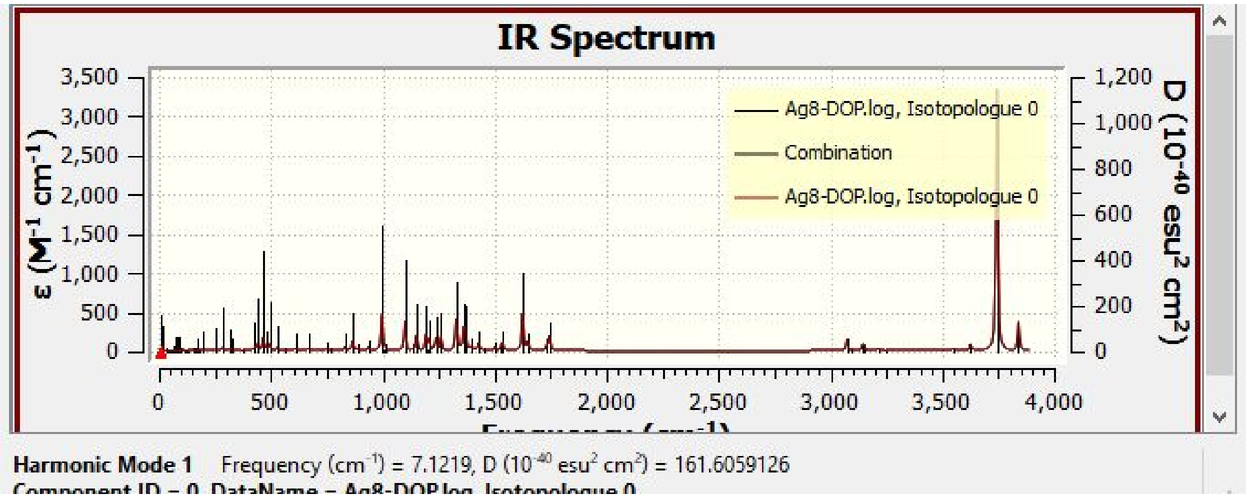

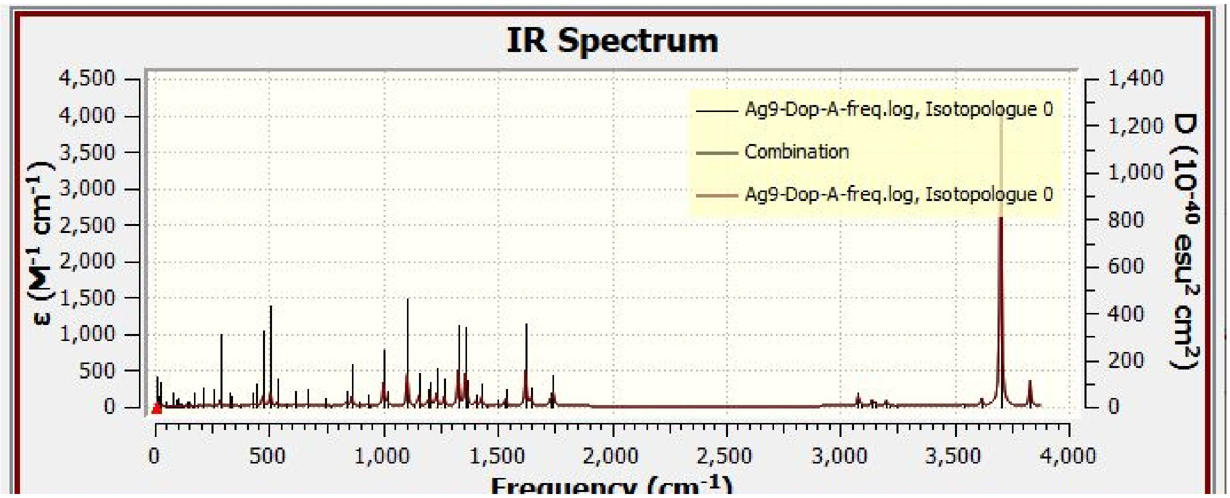

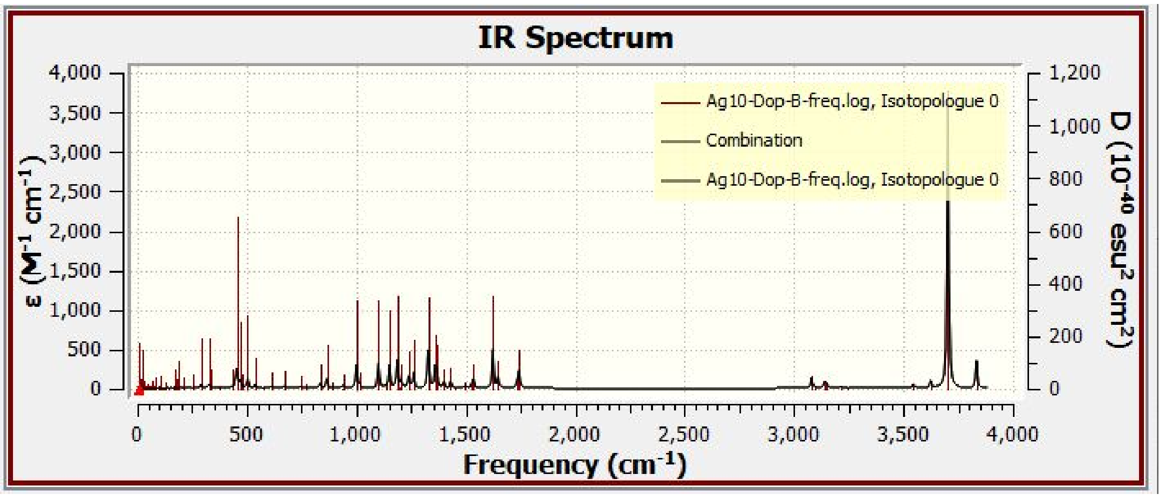

Fig. S1. *Continued.*

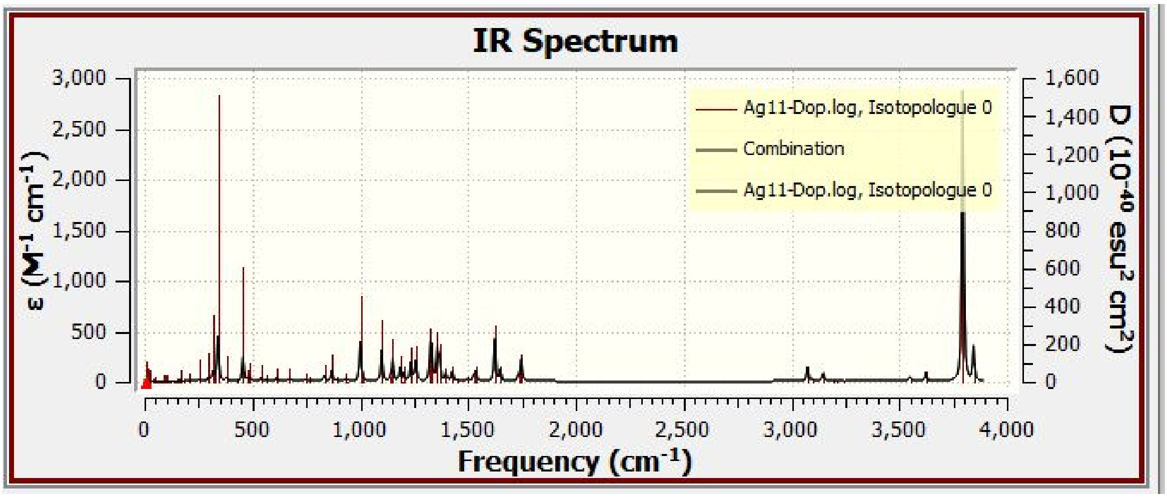

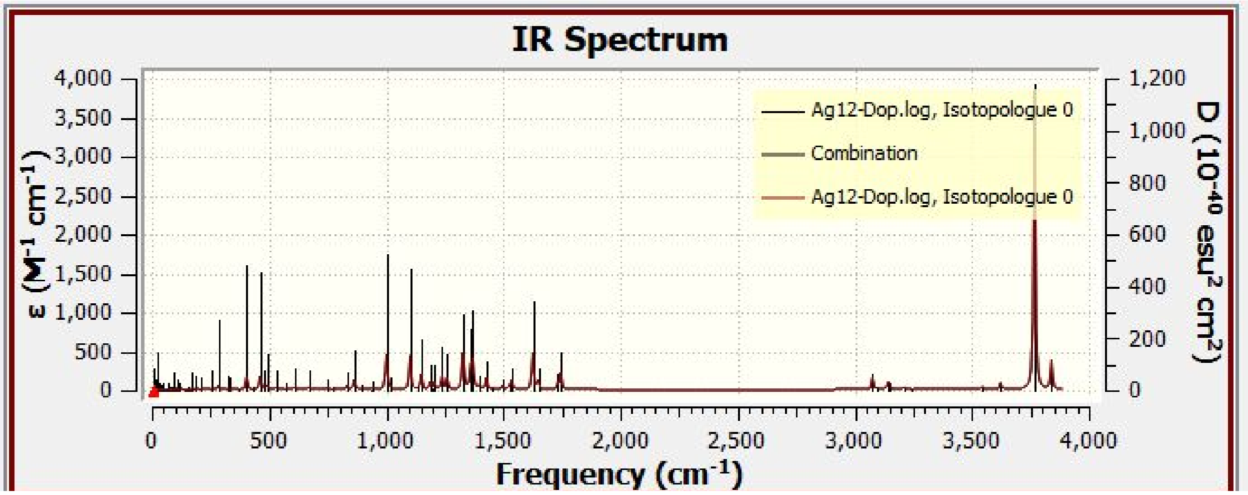

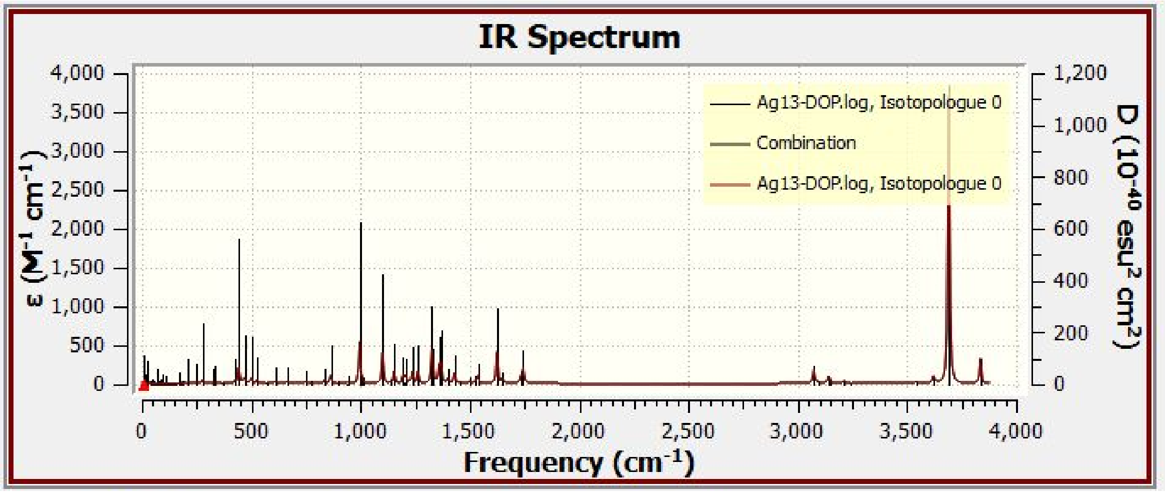

Fig. S1. *Continued.*

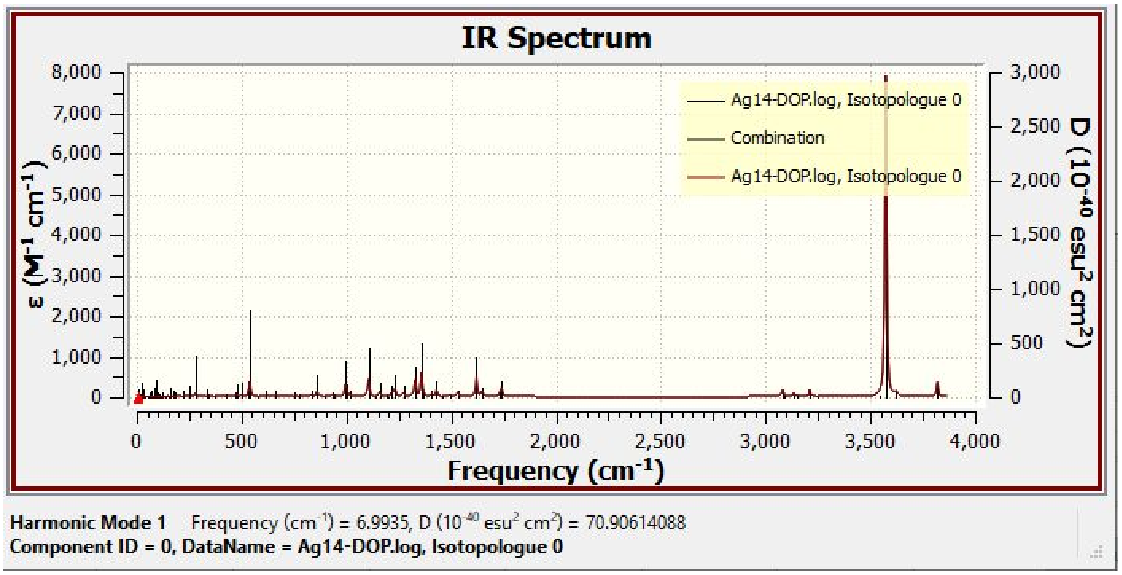

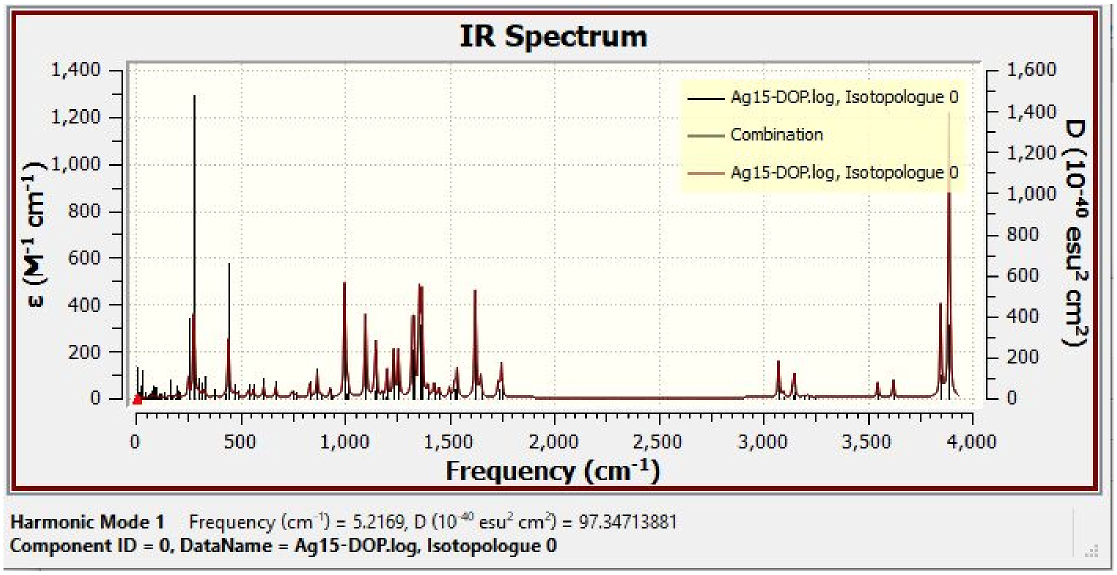

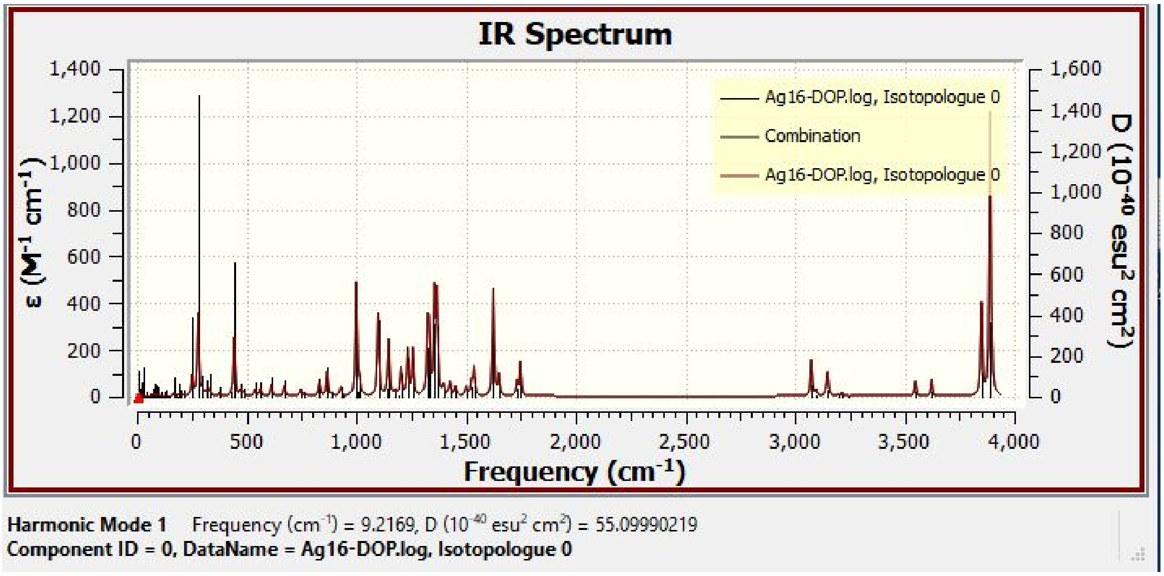

Fig. S1. *Continued.*

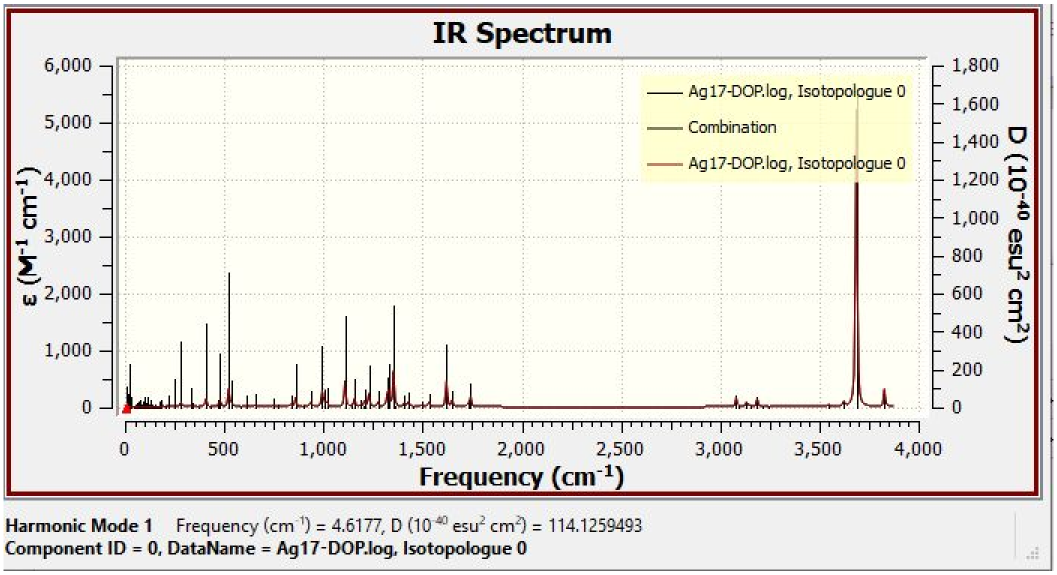

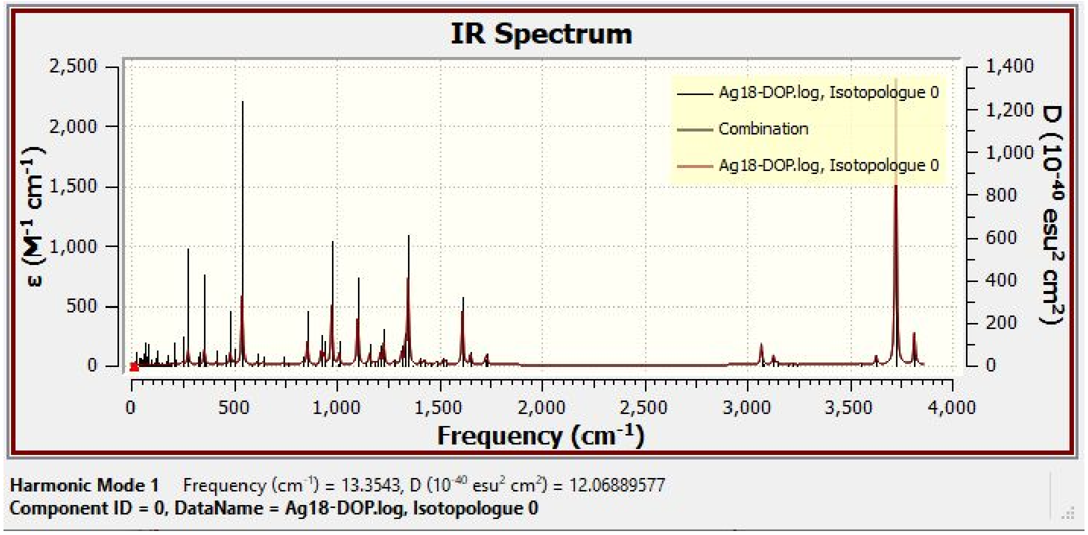

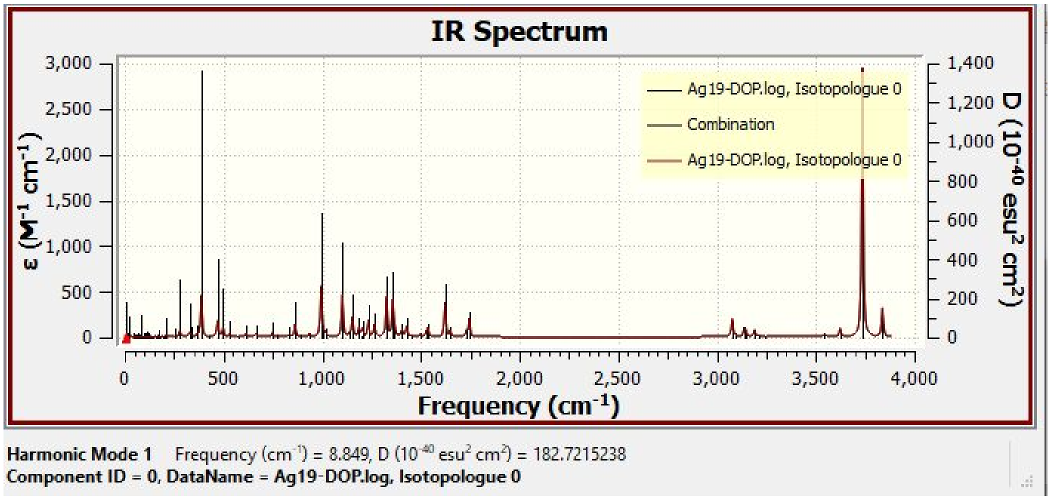

Fig. S1. *Continued.*

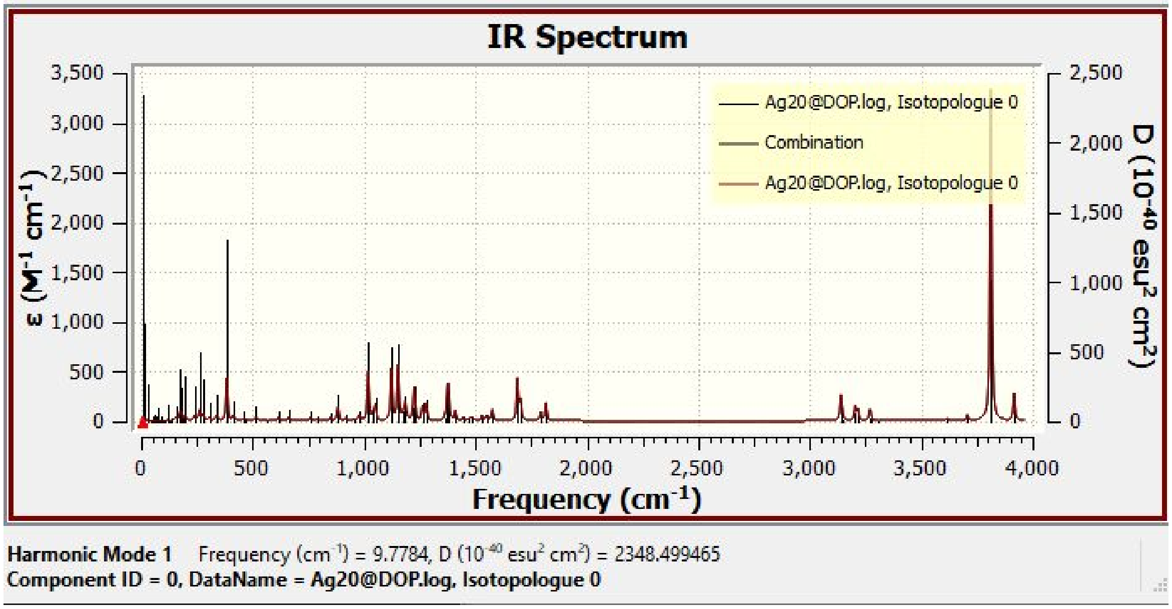

Fig. S1. *Continued.*
